# Supplementary material for: Actinium chelation and crystallization in a macromolecular scaffold
Source: Nat Commun. 2024 Jul 15;15:5741. doi: 10.1038/s41467-024-50017-5 (PMC11251196; doi:10.1038/s41467-024-50017-5)
Supplement: Supplementary file 1 — Supplementary Information [file 41467_2024_50017_MOESM1_ESM.pdf]

Supplementary Information for

Actinium Chelation and Crystallization in a Macromolecular Scaffold

Jennifer N. Wacker[1], Joshua J. Woods[1], Peter B. Rupert[2], Appie Peterson[1], Marc Allaire[3], Wayne W. Lukens[1], Alyssa N. Gaiser[1]†, Stefan G. Minasian[1], Roland K. Strong[2]*, Rebecca J. Abergel[1,4,5]*

Correspondence: abergel@berkeley.edu, rstrong@fredhutch.org

Table of Contents

1. Supplementary Methods 2
2. Supplementary Discussion 10
3. Supplementary Figures 13
4. Supplementary Tables 29
5. Supplementary References 34

1. Supplementary Methods

General Considerations

Caution! The ^{227}Ac isotope [$t_{½}$ = 21.772(3) years; 2.7 TBq·g^{-1}] and its daughter products, which generate alpha (α), beta (β), and gamma (γ) emissions, present severe radiological and toxicological hazards. As such, sample manipulations occurred in specialized radiological facilities equipped with administrative and engineering controls for sample handling, including certified HEPA-filtered fume hoods and appropriate α-, β-, and γ- detecting instruments for radiological work. Radiological workers donned appropriate personal protective equipment and dosimetry prior to any operations and performed personal and area surveys after experimentations. Furthermore, to minimize radiolysis effects and dose from the highly radioactive decay products, the ^{227}Ac samples were purified directly before measurements.

Unless otherwise specified, manipulations were performed under ambient temperatures and pressures and no efforts were made to exclude air and water. The following solvents and reagents were obtained commercially and used as received unless otherwise specified: 3,4,3-LI(1,2-HOPO) (HOPO) was obtained from Ash Stevens. Inc. as previously described,[1] ammonium sulfate ($(NH_4)_2SO_4$; VWR Chemicals BDH), aqueous solutions of hydrochloric acid ($HCl_{(aq)}$; 32–35%; OPTIMA™ Grade; Fisher Scientific), aqueous solutions of nitric acid ($HNO_{3(aq)}$; 67–70%; OPTIMA™ Grade; Fisher Scientific), ascarite CO_2 absorbent (ThermoFisher Scientific), europium ICP standard solution ($Eu_{(aq)}/HNO_3$; 1000 ppm Eu in 3% HNO_3; Ricca Chemical Company), glycerol ($C_3H_8O_3$; >99.5%; Sigma-Aldrich), lanthanum plasma standard solution ($La_{(aq)}/HNO_3$; 10,000 μg/mL; Specpure®; Alfa Aesar), lithium sulfate monohydrate ($Li_2SO_4·H_2O$; ACS, 99.0% min; Alfa Aesar), piperazine-N,N'-bis(2-ethanesulfonic acid) (PIPES; High Purity Grade; Amresco), potassium chloride (KCl; >99.5%, BioUltra, Sigma-Aldrich), potassium hydrogen phthalate (KHP; BioXtra 99.95%; Millipore Sigma), sodium acetate (CH_3COONa; ACS; VWR), sodium chloride (NaCl; Mallinckrodt), standardized solution of potassium hydroxide ($KOH_{(aq)}$; 0.1 M; BDH Chemicals), standardized solution of hydrochloric acid ($HCl_{(aq)}$; 0.1 M; BDH Chemicals), tris(hydroxymethyl)aminomethane (TRIS; Molecular Biology Grade Ultra Pure ≥ 99.9 %; JT Baker), ubiquitin (from bovin erythrocytes; BioUltra >98% (SDS-PAGE); Sigma-Aldrich). All water was deionized and passed through a Milli-Q® Direct water purification system prior to use at a resistivity of 18.2 MΩ · cm at 25 °C. Recombinant human siderocalin was prepared as previously described and stored in PN buffer at a concentration of approximately 10 mg/ mL.[2] An SDS-PAGE showing the quality of the final purified protein is presented in Supplementary Figure 22. The following resins were also obtained commercially in bottles and loaded into BioRad Poly-Prep® chromatography columns (10 mL) for use: pre-filter resin (100-150 μm; Eichrom), AG® 1-X8 resin (100-200 mesh; chloride form; BioRad), and DGA-Branched resin (100-150 mesh; Eichrom). Ultima Gold™ LSC cocktail (PerkinElmer, Inc.) was used for liquid scintillation counting (LSC). All crystallization solutions and buffers were sterile filtered (0.2 μm PES; Thermo Scientific) before use. Crystallizations were performed in NeXtal 15-well protein crystallization plates with standard crystallization supports. Upon crystallization, samples were manipulated under liquid nitrogen with cryo tools, magnetic mounts, and nylon cryoloops from Hampton Research. Figures were made with *GraphPad Prism* (version 10.0.2), *PyMOL Molecular Graphics System* (version 2.0, Schrödinger, LLC), *UCSF ChimeraX* (version 1.6.1),[3] and *Adobe Illustrator* (2023).

Instrumentation
Samples were calcined in a ThermoScientific Series 300 muffle furnace. Fluorescence measurements were collected on a PicoQuant FluoTime300 fluorescence lifetime and steady state spectrometer with customized measurement parameters controlled within the EasyTau 2 software. LSC measurements were taken on a Wallac 1414 Guardian liquid scintillation counter controlled with WinSpectral software (Perkin Elmer, Inc.), a Tri-Carb 2910 TR liquid scintillation counter (Perkin Elmer, Inc.), or a Tri-Carb 4910 TR liquid scintillation counter (Perkin Elmer, Inc.). Gamma (γ) spectroscopic measurements were performed on locally-built high purity germanium (HPGe) detector with a 16 mm diameter, n-type HPGE crystal and an aluminum-coated Kevlar window. The detector was calibrated using a mixed isotope standard (^{241}Am, ^{109}Cd, ^{57}Co, ^{139}Ce, ^{203}Hg, ^{113}Sn, ^{137}Cs, ^{88}Y, ^{60}Co and ^{152}Eu) traceable to the National Institute of Standards and Technology and supplied by Eckert & Ziegler. All γ-spectroscopic measurements were controlled with GammaVision software (Ortec) and analyzed using *Interspec* (version 1.0.9), a spectral radiation analysis software from Sandia National Laboratory, which accounted for the detector efficiency based on the distance of the sample to the detector.[4] Potentiometric titrations were performed on a Metrohm Titrando 888 titrator equipped with a Metrohm 806 exchange unit with an automatic burette (10 mL capacity), and an Orion 8103 Ross combination semi-micro pH electrode (8103BN, ThermoFisher Scientific). The entire device was controlled using Tiamo 3.0 software. UPLC-MS measurements were acquired using a UPLC Waters Xevo system interfaced with a QTOF mass spectrometer (Waters Corporation) in Micromass Z-spray geometry. UV-visible spectra were collected on a Varian Cary 5G UV-Vis-NIR spectrophotometer.

Experimental Details
Preparation of actinium stock solution.
The ^{227}Ac stock was recovered from samples used for other studies, which had themselves been extracted from a legacy protactinium-231 [^{231}Pa; $t_{½}$ = 3.276(11) x 10^4 y] source at the Lawrence Berkeley National Laboratory. After several decades, there was substantial daughter product in-growth in the ^{231}Pa stock. The decay products, namely ^{227}Ac^{III}, had been leached from the solid ^{231}Pa$^{V}{}_2$O$_5$ with nitric acid and used in previous studies. These ^{227}Ac^{III} samples were aggregated, reprocessed, and used in the studies reported herein. The reprocessing of these samples to generate a chemically and radiochemically pure ^{227}Ac^{III} stock was performed according to previous reports.[5, 6, 7, 8] Briefly, legacy ^{227}Ac^{III} samples were aggregated to yield a slurry; most of the sample mass arose from chromatography resins that had been loaded with ^{227}Ac^{III} and daughters from past experiments. The ^{227}Ac^{III} slurry was first calcined in quartz containers to remove organic contaminants. The slurry was held to 85 °C overnight to remove residual water. Thereafter, the temperature was ramped to 330 °C at a rate of 1 °C / minute and held for 46 hours. Finally, the temperature was ramped to 1000 °C at a rate of 1 °C / minute and held for 30 minutes, then slow-cooled for 15 hours. The quartz containers were rinsed with $HNO_{3(aq)}$ (8 M, ~50 mL) and the solution was transferred to a polyethylene Falcon cone (50 mL). Chemical contaminants not removed during calcination, along with ^{227}Ac^{III} daughter products, were separated through four additional steps. First, the crude ^{227}Ac^{III} solution in $HNO_{3(aq)}$ (8 M, ~50 mL) was run through pre-filter resin (1 mL; conditioned with (i) 3 ×5 mL H_2O and (ii) 3 × 5 mL 8 M $HNO_{3(aq)}$) to remove trace amounts of organic impurities. The volume was reduced overnight to ~15 mL. Next, the crude ^{227}Ac^{III} solution in $HNO_{3(aq)}$ (8 M, ~15 mL) was loaded on to another column to remove the thorium-227 (^{227}Th^{IV}) daughter by anion exchange chromatography with AG® 1-X8 resin (1 mL;

conditioned with (i) 3 × 5 mL H_2O, (ii) 3 × 5 mL 8 M $HNO_{3(aq)}$, (iii) 3 × 5 mL H_2O, and (iv) 3 × 5 mL 8 M $HNO_{3(aq)}$). The eluent, which contained $^{227}Ac^{III}$ and radium-223 ($^{223}Ra^{II}$) radionuclides, was diluted to 6 M $HNO_{3(aq)}$ before being loaded onto DGA resin, branched (DGA-B, *N,N,N',N'*-tetrakis-2-ethylhexyldiglycolamide) to remove the $^{223}Ra^{II}$ daughter by extraction chromatography (1 mL; conditioned with (i) 3 × 10 mL H_2O and (ii) 1 × 10 mL 6 M $HNO_{3(aq)}$; capped with a frit). The $^{223}Ra^{II}$ daughter passed through the column whereas $^{227}Ac^{III}$ was retained. The column was washed with $HNO_{3(aq)}$ (6 M; 6 × 2 mL). The $^{227}Ac^{III}$ was eluted with $HNO_{3(aq)}$ (0.05 M; 2 × 5 mL). Lastly, the $^{227}Ac^{III}$ solution was run through a second pre-filter column (1 mL; conditioned with (i) 3 × 5 mL H_2O and (ii) 3 × 5 mL 0.05 M $HNO_{3(aq)}$) to remove trace amounts of organic impurities introduced from the DGA-B resin. This final solution yielded a radiochemically pure stock of $^{227}Ac^{III}$. The $^{227}Ac^{III}$ solution in 0.05 M $HNO_{3(aq)}$was brought to a residue overnight under slight heat and an air stream and used immediately the following day for experiments. This process was repeated partially (without calcination) or wholly (with calcination) multiple times throughout these studies to ensure the purity of the $^{227}Ac^{III}$ stock. A visual representation of this process can be viewed in Supplementary Figure 1.

Quantification of actinium.
The quantification of ^{227}Ac is particularly difficult due to the rapid ingrowth of daughter products, the β- and γ- emissions, which could be useful for quantification with LSC and γ-spectroscopy, but are relatively low intensities, and the limited ^{227}Ac mass quantities available. Because of these issues, a more rigorous approach to quantify ^{227}Ac would be through the Bateman equation by monitoring the ingrowth of the daughter products.[8, 9] However, the studies reported herein aimed to tease out the chemistry of ^{227}Ac alone with techniques that do not have the luxury of element specificity, like X-ray Absorption Spectroscopy. As such, the studies required ^{227}Ac purification immediately before the experiment began. Therefore, concessions on quantification techniques were warranted in order to meet this requirement, as ^{227}Th and ^{223}Ra ingrowths were not appreciable within 1–12 hours after separation to reliably employ the Bateman equation. Instead, our approach to ^{227}Ac quantification included both LSC and γ-spectroscopy. LSC directly probed ^{227}Ac β-emission while γ-spectroscopy indirectly probed ^{227}Ac with γ-emissions from daughter ingrowth. Exemplary spectra from these measurements are provided in Supplementary Figure 3. Both approaches have their disadvantages, which are outlined below.

LSC can directly quantify the activity of ^{227}Ac; however, there are several drawbacks of using this technique that are specific to this radionuclide. LSC requires dissolution of the sample (≤0.1 μCi for accurate activity determination) in a scintillation cocktail. Therefore, recovery of the material is practically infeasible, limiting the use of this quantification method. Furthermore, while all of the LSC measurements in this study were performed using alpha/beta discrimination, this approach includes a small degree of imprecision (typically <1%) due to misidentification of alpha and beta decays. The maximum energy of ^{227}Ac β-decay is 44.8(8) keV (Q_{decay} value, National Nuclear Data Center), which is in a low energy regime wherein the detection efficiency of the LSC detector is not 100%. We used a detector efficiency of 84% for ^{227}Ac calculations.[10] In addition, ^{223}Fr β-emission (Q_{decay} = 1149.1(9) keV) overlaps slightly with the ^{227}Ac β-emission to further introduce inaccuracies. The overlap was addressed by binning the β-emission region so that the ^{227}Ac and ^{223}Fr overlap was essentially equal (bin regions: ^{227}Ac = 0–45 keV, ^{223}Fr = 45–1100 keV, alphas = 200–1000 keV).

γ-spectroscopy does not alter the integrity of the sample (no dissolution in a scintillation cocktail required), but relies solely on daughter emission lines. The primary daughter product in

the ^{227}Ac decay chain is ^{227}Th [$t_{1/2}$ = 18.697(36) days, branching ratio = 98.62%], which would require approximately 112 days or 6 half-lives in order to reach secular equilibrium to accurately quantify ^{227}Ac. As such, the ^{223}Fr daughter [$t_{1/2}$ = 22.00(7) minutes, branching ratio = 1.38%] can be used as a fingerprint instead, with secular equilibrium reached in *ca.* 2.2 hours. By using the ^{223}Fr γ-line at 50.094(15) keV and subtracting out any contribution from ^{227}Th ingrowth at 50.13(1) keV, the ^{227}Ac concentration can theoretically be calculated. However, quantification *via* the low energy ^{223}Fr γ-line is impaired by the detector efficiency and further inhibited by the overlapping ^{223}Fr / ^{227}Th γ-lines, and therefore this method was used as a supplementary check to LSC activity determinations.

In summary, quantification of ^{227}Ac is uniquely challenging and therefore, we acknowledge the intrinsic error associated with ^{227}Ac concentrations reported in these experiments. Nonetheless, we approached the inherent issues in a manner best fit for the instrumental limitations and time constraints at hand. Other methodologies have also been reported to accurately quantify ^{227}Ac with highly specialized methods, but are outside the scope of this work.[10, 11, 12]

Potentiometric titrations.

The protonation constants of HOPO were determined potentiometrically. The water-jacketed titration vessel was fitted with a removable glass insert (~50 mL volume) and was maintained at 25 °C using a circulating water bath. Standardized solutions of $KOH_{(aq)}$ and $HCl_{(aq)}$ (0.1 M) were obtained commercially, and their exact concentrations were determined by potentiometric titration against KHP or TRIS base, respectively. Standardized $KOH_{(aq)}$ was stored in polyethylene bottles under a small positive pressure of argon and the bottle was fitted with a plastic tube filled with ascarite CO_2 absorbent to prevent dissolution of atmospheric $CO_{2(g)}$. The titration vessel was maintained under a small positive pressure of argon scrubbed with 30 wt% $KOH_{(aq)}$.

Before each titration, the glass electrode was calibrated in terms of H^{1+} concentration by titrating a solution of $HCl_{(aq)}$ (5 mM) with standardized $KOH_{(aq)}$. The ionic strength was maintained at 0.5 M using $KCl_{(aq)}$, and the titration solution equilibrated for 10 minutes prior to addition of the titrant. Data within the pH ranges of 2.5–3.2 and 10.8–11.3 were analyzed using the program *Glee* (version 3.0.21)[13] to obtain the standard electrode potential (E_0) and slope factor. The H_2O ion product of pK_w = 13.72 was used.[14] The presence of adventitious $CO_{2(g)}$ in the $KOH_{(aq)}$ solution was determined using Gran's method[15] and was below 1.5% for all titrations. The ligand protonation constants were measured by adding standardized $KOH_{(aq)}$ (20 μL aliquots) to an aqueous solution (~20 mL) containing ligand (0.5–1 mM) and $HCl_{(aq)}$ (5 mM). The ionic strength of the solution was maintained at 0.5 M using $KCl_{(aq)}$. The titration method employed a 0.1 mV min^{-1} drift limit with a maximum wait time of 180 seconds between base additions. The ligand concentration was estimated by the two endpoints of the titration curve.

The protonation constants were determined using the program *Hyperquad2013*.[16] The proton and ligand concentrations were admitted as refinable parameters, and the refined ligand concentration varied by less than 10% from what was determined as described above. The protonation constants were determined using at least two independently prepared stock solutions of ligand and are reported as the average of three independent titrations with ≥ 65 data points over the pH range of 2.5–11 for each replicate.

Fluorescence competition semi-batch assays.
All experiments were performed in Starna Cell screw-cap cuvettes for small volumes (700 μL) made from Spectrosil® Quartz. Measurements were collected in steady-state mode with a Xenon arc lamp (300 W) excitation source. Spectral selection was achieved by passage through a double grating excitation monochromator (2.7 nm/mm dispersion, 1200 grooves/mm, 100% attenuation). Emission was monitored perpendicular to the excitation pulse with spectral selection achieved by passage through a double grating emission monochromator (2.7 nm/mm dispersion, 1200 grooves/mm). Excitation and emission spectral slit widths varied based on the Eu^{III} concentration, ranging from 2-8 mm. Data were collected with a UV/VIS photomultiplier tube detector capable of single photon counting. All measurements were collected at 25 °C, maintained using a circulating water bath within the spectrometer sample chamber.

The conditional formation constants (log β'_{ML}) of $[La^{III}(HOPO)]^{1-}$ and $[Ac^{III}(HOPO)]^{1-}$ were determined by spectrofluorometric competition titrations against europium as previously described.[17] General experimental methods were followed for all competition titrations. The solutions were buffered in 0.1 M TRIS (pH 7.36) and the ionic strength of the solution was maintained at 0.5 M with $KCl_{(aq)}$. Control experiments with La^{III} verified that the solution was effectively buffered so that the pH remained constant despite $La^{III}/HNO_{3(aq)}$ additions at pH 7.36. For $^{227}Ac^{III}$ measurements, the pH of the solution was checked at the beginning and end of the titration by spotting an aliquot (1 μL) on pH paper and was confirmed to remain constant throughout the course of the experiment. After each titration addition, the solutions were equilibrated by agitation for 1 hour at 25 °C using an orbital shaker before the fluorescence spectrum of the sample was acquired. Control experiments with La^{III} provided similar values for log β'_{ML} if the solutions were allowed to equilibrate for 1 hour or 48 hours, suggesting that a 1-hour equilibration period was sufficient to achieve equilibrium (see Supplementary Table 1). Additional control experiments that considered buffering conditions, batch vs. semi-batch approaches, and europium starting concentrations were also performed and summarized in Supplementary Table 1. Similar log β'_{ML} values for $[La^{III}(HOPO)]^{1-}$ were observed regardless of these varying conditions, and thus provided experimental validation of to extend this approach to $^{227}Ac^{III}$ thermodynamic studies. The log β'_{ML} values reported are the average of triplicate measurements with error included as the standard deviation of the mean value. Following are the experimental methods for those measurements presented in the main text (Figure 2).

3 μM $[Eu^{III}(HOPO)]^{1-}$: The conditional formation constant (log β'_{ML}) of $[La^{III}(HOPO)]^{1-}$ was determined by spectrofluorometric competition titrations against europium using a starting concentration of 3 μM $[Eu^{III}(HOPO)]^{1-}$. In a screw-capped 1 cm quartz cell, aliquots (2.5 μL) of the La^{III} stock solution [60 mM in 0.005 mM $HNO_{3(aq)}$] were added to a solution of ligand (3 μM) and europium (3 μM) in 0.1 M TRIS (pH 7.36) with the ionic strength of the solution maintained at 0.5 M with $KCl_{(aq)}$. One hour after each addition, the fluorescence spectrum (λ_{ex} = 325 nm, λ_{em} = 570 nm–630 nm) of the sample was acquired.

50 nM $[Eu^{III}(HOPO)]^{1-}$: The conditional formation constants (log β'_{ML}) of $[La^{III}(HOPO)]^{1-}$ was determined by spectrofluorometric competition titrations against europium using a starting concentration of 50 nM $[Eu^{III}(HOPO)]^{1-}$. In a screw-capped 1 cm quartz cell, aliquots (2.5 μL) of the La^{III} stock solution [1 mM in 0.05 mM $HNO_{3(aq)}$] were added to a solution of ligand (50 nM) and europium (50 nM) in 0.1 M TRIS (pH 7.36) with the ionic strength of the solution maintained at 0.5 M with $KCl_{(aq)}$. One hour after each addition, the fluorescence spectrum (λ_{ex} = 325 nm, λ_{em} = 570 nm–630 nm) of the sample was acquired.

10 nM [Eu^{III}(HOPO)]$^{1-}$: The conditional formation constants (log β'_{ML}) of [La^{III}(HOPO)]$^{1-}$ and [Ac^{III}(HOPO)]$^{1-}$ were determined by spectrofluorometric competition titrations against europium using a starting concentration of 10 nM [Eu^{III}(HOPO)]$^{1-}$. Varying volumes (2.5–5 μL) of La^{III} or $^{227}Ac^{III}$ stock solutions [100 μM in 0.05 mM $HNO_{3(aq)}$] were added to a solution of ligand (10 nM) and europium (10 nM) in 0.1 M TRIS (pH 7.36) in the screw capped 1 cm quartz cell and equilibrated for 1 hour. The $^{227}Ac^{III}$ stock was purified (see *methods: purification of actinium stock solution)* 12 hours before log β'_{ML} measurements were performed. The ionic strength of the solution was maintained at 0.5 M with $KCl_{(aq)}$. After each addition, the fluorescence spectrum (λ_{ex} = 325 nm, λ_{em} = 570 nm–630 nm) of the sample was acquired. In the $^{227}Ac^{III}$ titrations, the concentration of the $^{227}Th^{IV}$ daughter in the cuvette was quantified by γ-spectroscopy using the 235.96 keV line (11.2% intensity) after each $^{227}Ac^{III}$ addition.

For analyses, the data were imported into the refinement program *HypSpec* and analyzed by nonlinear least-squares refinement. All metal and ligand concentrations were held constant at the estimated concentrations from their standardized stock solution. The refinement of the overall formation constant included in each case the four ligand protonation constants, the metal hydrolysis constants, the previously determined log β_{ML} value for Eu^{III},[17] and the luminescence spectra of [Eu^{III}(HOPO)]$^{1-}$. The β_{MLH} value for Eu^{III} was not included in the model. Initial attempts to fit the data with this formation constant included did converge, but upon closer investigation we found that the predicted molar spectrum of the MHL species to be 1000 times more intense than the ML species, which is physically unreasonable. Additionally, the refined speciation diagram suggested that the MHL species to make up <0.05% of the total Eu^{III}-containing species in solution at pH 7.36. As such, the data were fit without the inclusion of this species. The log β_{ML} for [$^{232}Th^{IV}$HOPO] (40.1)[18] and the $^{232}Th^{IV}$ hydrolysis constants[19] were also included in the actinium titrations to account for the presence of the $^{227}Th^{IV}$ daughter as determined by γ-spectroscopy, collected throughout the experiment after each fluorescence spectrum was acquired. The log β'_{ML} values reported are the average of triplicate measurements with error included as the standard deviation of the mean value. This method was used to determine the conditional formation constants (log β'_{ML}) of [La^{III}(HOPO)]$^{1-}$ and [Ac^{III}(HOPO)]$^{1-}$ according to the following equations:

$$m\mathrm{M} + l\mathrm{L} + h\mathrm{H} \leftrightarrow [M_m L_l H_h] \qquad \text{(Supplementary Equation 1)}$$

$$\beta'_1 = \frac{[M_m L_l H_h]}{[M]^m [L]^l [H]^h} \qquad \text{(Supplementary Equation 2)}$$

Fluorescence quenching assays.
All instrumental and experimental considerations were identical to the *fluorescence competition semi-batch assays* noted above. One additional consideration should be noted: the excitation shutter was opened immediately before and closed immediately after spectral measurements to limit photobleaching of the protein. Emission spectra (λ_{ex} = 281 nm) were collected from 338–345 nm (slit sizes = 2.96 mm; integration time per point = 0.3 s; step size, Δ = 0.5 nm). The limited spectral range was imposed to also limit photobleaching of the protein. Concentrations were corrected for dilution upon addition of the titrant.

The dissociation constants (K_{D}) were measured by fluorescence quenching assays according to previously reported methods.[20] The K_{D} values were determined for siderocalin with (i) HOPO, (ii) La^{III}, and (iii) $^{227}Ac^{III}$. First, the ligand or metal-ligand quenching solutions were prepared. A stock solution of HOPO was made by dissolving a weighted portion of the ligand into

Milli-Q water (10.26 mM; concentration determined potentiometrically). A working solution of HOPO (10.26 mM) was further diluted with TBS buffer (25 mM TRIS and 100 mM NaCl; pH 7.4) to yield a final concentration of 1 μM for ligand titrations. Metal complexes were formulated by combining portions of the stock solution of HOPO with the M^{III} ion (M^{III} = La^{III}, $^{227}Ac^{III}$) in 0.05 M $HNO_{3(aq)}$ to yield a metal:ligand molar ratio of 1:1. Specifically, $[La^{III}(HOPO)]^{1-}$ was prepared by diluting La^{III} from a lanthanum inductively coupled plasma (ICP) standard solution ($La_{(aq)}/HNO_3$; 10,000 μg/mL) into 0.05 M $HNO_{3(aq)}$ to yield a final solution of 0.05 mM, while the solution of HOPO (10.26 mM) was further diluted to yield a final concentration of 0.1 mM. From these working solutions, La^{III} in $HNO_{3(aq)}$ (0.05 mM; 100 μL; 50 nmol; 6.95 μg) was added to HOPO (0.1 mM; 50 μL; 50 nmol; 37.54 μg) and equilibrated for 1.5 hours. Thereafter, TBS buffer (25 mM TRIS and 100 mM NaCl; pH 7.4) was added to yield a final concentration of 1 μM and equilibrated overnight. Similarly, $[Ac^{III}(HOPO)]^{1-}$ was synthesized by combining working solutions of $^{227}Ac^{III}$ in 0.05 M $HNO_{3(aq)}$ and $HOPO_{(aq)}$. An important difference is that $^{227}Ac^{III}$ was purified (see *methods: purification of actinium stock solution)* 48 hours before K_D measurements were performed. The freshly purified stock of $^{227}Ac^{III}$ in $HNO_{3(aq)}$ (100 μM; 16.02 μL) was further diluted with $HNO_{3(aq)}$ (0.05 M; 800 μL) to yield a working stock solution of $^{227}Ac^{III}$ in $HNO_{3(aq)}$ (2 μM). The solution of HOPO (10.26 mM) was further diluted to yield a final concentration of 0.11 mM. From these working solutions, $^{227}Ac^{III}$ in $HNO_{3(aq)}$ (2 μM; 100 μL; 50 nmol; 6.95 μg) was added to HOPO (0.1 mM; 50 μL; 50 nmol; 37.54 μg) and equilibrated for one hour. Thereafter, TBS buffer (25 mM TRIS and 100 mM NaCl; pH 7.4) was added to yield a final concentration of 1 μM and equilibrated for an additional hour. Collectively, these working solutions served as the titrant in the fluorescence quenching assays.

Separately, in Eppendorf™ LoBind Microcentrifuge tubes (1.5 mL), siderocalin (diluted to 50 μM; 1 μL) was added to TBS (500 μL; 25 mM TRIS and 100 mM NaCl; pH 7.4) containing ubiquitin (32 μg / mL) and transferred to quartz cells to yield an initial protein concentration of *c.a.*100 nM. Additions of HOPO or $[M^{III}(HOPO)]^{1-}$ complexes were repeatedly added to the cuvette (10 nM additions), the samples were inverted 5 times, equilibrated for at least 5 minutes, and emission spectra were collected. The intensities at λ_{em} = 340 nm were tabulated, normalized, and fitted with the *DynaFit* software program to calculate dissociation parameters.[21] The K_D values reported are the average of three trials and the error is the standard deviation of the mean (Supplementary Tables 4–6). The K_D values were determined according to the following equations:

$$\mathrm{Scn} + \mathrm{ML} \leftrightarrow [\mathrm{Scn{:}\,ML}] \qquad \text{(Supplementary Equation 3)}$$

$$\beta_2' = K_\mathrm{A} = \frac{1}{K_\mathrm{D}} = \frac{[\mathrm{Scn{:}ML}]}{[\mathrm{Scn}][\mathrm{ML}]} \qquad \text{(Supplementary Equation 4)}$$

Macromolecular crystallography: sample preparation, crystallization, data collection / analysis. Immediately after the ^{227}Ac purification described above, the ^{227}Ac residue was dissolved in $HNO_{3(aq)}$ (0.05 M; 20 μL) to yield an ^{227}Ac concentration of 1 mM (21.5 nmol; ~4.88 μg). The solution was transferred to an Eppendorf™ LoBind Microcentrifuge tube (1.5 mL) containing HOPO dissolved in DMF (10 mM; 2.20 μL; 22.01 nmol; 16.52 μg) to yield a metal:ligand ratio of 1:1.1 and the solution was equilibrated for one hour. Then, the solution was buffered with TRIS buffer (1 M; pH 8; 50 μL) was added to the solution and diluted with water (358 μL) to generate a final TRIS concentration of 116 mM. This solution equilibrated at room temperature for one hour. Human siderocalin (Scn) was added in a metal–ligand:protein ratio of approximately 1:2

(0.486 mM; 82.3 μL; 40 nmol; 821.9 μg) and the solution was equilibrated for 30 minutes. Thereafter, the entirety of this solution was transferred to a molecular weight cutoff spin filter (0.5 mL; 10 K cutoff; Thermo Scientific Pierce™ Concentrator, PES) and centrifuged (10,000 x g; 10 minutes) to purify the ^{227}Ac–HOPO–Scn construct. The retentate was buffer exchanged into PIPES buffer (25 mM; 2 x 200 μL; pH 7.4) by further centrifugation to yield a solution (*ca.* 50 μL) of the ternary ^{227}Ac–HOPO–Scn construct at a protein concentration of approximately 10 mg/mL. The ternary ^{227}Ac–HOPO–siderocalin complex was crystallized via the hanging drop method as previously reported.[20] The protein drop consisted of 2 μL protein solution and 2 μL reservoir solution. The components of the reservoir solutions included $Li_2SO_{4(aq)}$ (200 mM), $NaOAc_{(aq)}$ (100 mM; pH 3.9–4.3), $NaCl_{(aq)}$ (50 mM), and $(NH_4)_2SO_{4(aq)}$ (1.25–1.45 M). The drop was equilibrated against 975 μL of the corresponding reservoir solution. Diffraction quality crystals were grown over the course of one week (Supplementary Figure 16).

The ternary La–HOPO–siderocalin complex was crystallized using similar methods as described above. The main differences between La and Ac crystallizations were (1) the concentration of metal–ligand complex could be in excess compared to the protein concentration as is typical for protein crystallizations (La–HOPO:protein ratio was approximately 2:1 vs. the Ac–HOPO:protein ratio of approximately 1:2) and (2) the length of crystallization time was not an issue as there would be no radiolytic damage present as compared to the Ac system (Supplementary Figure 16). A stock solution of La^{III} (10 mM) in $HNO_{3(aq)}$ (0.05 M) was made by dissolution of a lanthanum ICP standard solution ($La_{(aq)}/HNO_3$; 10,000 μg/mL). La^{III} in $HNO_{3(aq)}$ (10 mM; 5 μL; 50 nmol; 6.95 μg) was combined with HOPO dissolved in DMF (10 mM; 5.1 μL; 51 nmol; 38.2 μg) to yield a metal:ligand ratio of 1:1.1 and equilibrated at room temperature for one hour in an Eppendorf™ LoBind Microcentrifuge tube (1.5 mL). Then, TRIS buffer (100 mM; pH 8; 400 μL) was added to yield a final volume of 410.1 μL. The solution equilibrated at room temperature for one hour and then the pH was confirmed to be approximately pH 8 with pH paper. Human siderocalin was added to the solution for a La–ligand: protein ratio of approximately 2:1 (0.406 mM; 50 μL; 20.3 nmol; 415.3 μg) and equilibrated for 30 minutes. The entirety of this solution was transferred to a molecular weight cutoff spin filter (0.5 mL; 10 K cutoff; Thermo Scientific Pierce™ Concentrator, PES) and centrifuged (10,000 x g; 10 minutes) to purify the La–HOPO–Scn construct. The retentate was washed with PIPES buffer (25 mM; 2 x 200 μL; pH 7) by further centrifugation to yield a solution (*ca.* 50 μL) of the ternary La–HOPO–Scn construct at a protein concentration of approximately 10 mg/mL. Thereafter, diffraction quality crystals were grown via the hanging drop method under the same crystallization conditions used for Ac^{III} over the course of two months (Supplementary Figure 16).

Prior to analysis at the Advanced Lightsource (ALS), crystals were transferred to a new solution containing the same buffer concentrations used for crystallization with the addition of 20% v/v glycerol, looped on Hampton Research magnetic mounts with CryoLoops™, cryopreserved in liquid nitrogen, and loaded into an ALS-style puck. The nonradioactive La samples were loaded onto the goniometer automatically by the automounter and datasets were collected through automated programming. Given the radioactive nature of the Ac samples, the beamline was posted as a temporary contamination area, all samples were hand-mounted onto the goniometer by a researcher wearing personal protective equipment, contamination survey instruments were present, a protective layer of aluminum foil covered the area around the goniometer, and post-work surveys were performed to ensure no contamination was present after the measurements.

X-ray diffraction data (T = 100 K) were collected at beamline 5.0.2 at the ALS. For both the Ac and La structures, initial phases were obtained using Refmac[22] as implemented in the CCP4 software suite[23] by rigid body refinement using the coordinate set 1L6M.pdb as the initial starting model. Iterative rounds of alternating positional refinement and model building, using the programs Refmac[22] and COOT,[24] including placement of ordered solvent molecules and sulfate ions, were followed by a final round of TLS refinement.[25] Residues or side-chains that did not exhibit $2F_{obs}$–F_{calc} electron density were contoured at 0.7σ were removed or truncated to the $C\beta$ atom. The quality of the final model was assessed using *ProCheck*[26] and *Molprobity*.[27] The final models have been deposited in the Protein Data Bank[28] with accession codes 8UZ9 and 8UYN for the Ac and La structures, respectively. Crystallographic statistics are reported in Supplementary Table 7.

Additional characterizations: ultra-high-performance liquid chromatography-mass spectrometry (UPLC-MS) and absorption spectroscopy.
UV-vis traces were monitored between 200–400 nm. The UPLC system was fitted with an ACQUITY UPLC BEH C18 column (particle size: 1.7 μm, column size: 2.1 x 50 mm). Methods were performed using a flow rate of 0.5 $mL \cdot min^{-1}$ with a binary mobile phase consisting of (A) H_2O + 0.1% formic acid and (B) CH_3CN + 0.1% formic acid using the following gradient: 0 – 1 min 5% B, 1 – 3 min linear gradient to 100% B, 3 – 3.5 min 100% B, 3.5 – 4 min linear gradient to 5% B, 4.5 – 5 min 5% B. Mass spectra were acquired in the continuum mode across the m/z range of 100−1500 at 0.5 s per scan. The operating parameters were as follows: the nebulization gas flow rate was set to 800 L/h with a desolvation temperature of 300 °C, the cone gas flow rate was set to 1 L/h, and the ion source temperature was 120 °C. The capillary, sampling cone, and extraction cone voltages were tuned to 3 kV, 40 V, and 4 V, respectively. Liquid nitrogen served as a source of nebulizer gas. Data acquisition and instrument control were accomplished using *MassLynx* software (version 4.1). Samples were infused into the ionization chamber from the UPLC system. All mass spectra were referenced internally using leucine encephalin (2 ng/μL) as the lockspray. The instrument was calibrated periodically using sodium formate following manufacturer's instructions. Additional UV-vis traces were performed on a UV-vis spectrophotometer in a screw capped 1 cm quartz cell at higher concentrations of ligand/metal to replicate the UV-vis traces monitored by the UPLC system.

2. Supplementary Discussion

Experimental approach and validation of actinium stability constants through surrogate experiments with lanthanum.
Fluorescence competition titrations with lanthanum served as control experiments in preparation for measurements with $^{227}Ac^{III}$. In general, the consistency of log β'_{ML} values across various conditions from competition titrations with La^{III} and $[Eu^{III}(HOPO)]^{1-}$ (i.e., metal concentration, batch titration, equilibration period, slit size, buffer conditions) support the experimental approach and log β'_{ML} value reported for $^{227}Ac^{III}$. The experimental methods were analogous to those outlined in the *fluorescence competition semi-batch assays* methods section, with purposeful changes to one specified experimental variable. These tested conditions are compared to reference conditions and summarized in Supplementary Table 1. An example of emission spectrum of $[Eu^{III}(HOPO)]^{1-}$ as a function of slit size is provided in Supplementary Figure 4. UPLC-MS and

absorption spectroscopic measurements of the $[Eu^{III}(HOPO)]^{1-}$ complex in 0.1 M TRIS (pH 7.36) with the ionic strength of the solution maintained at 0.5 M with $KCl_{(aq)}$ are presented in Supplementary Figures 5–7.

Characterization of the lanthanum-HOPO complex as a surrogate for the actinium-HOPO complex in fluorescence quenching assay measurements.

Due to limited mass quantities and radiological safety considerations, the complexation of actinium with HOPO could not be characterized by UPLC-MS and absorption spectroscopy. Instead, the conditions that the actinium complex was formed were examined using a surrogate to assess the actinium chelation. Although it is likely that actinium will generally behave like its non-radioactive lanthanide surrogate based on their similar ionic radii and complexation behaviors,[29] it should be noted that this is not a direct measurement of actinium, but instead was used to provide supporting evidence for the complexation of actinium by HOPO.

The $[La^{III}(HOPO)]^{1-}$ complex was formed by combining an aliquot of La^{III} in $HNO_{3(aq)}$ (10 mM; 5 μL; 50 nmol; 6.95 μg) with HOPO dissolved in DMSO (10.1 mM; 5 μL; 50.5 nmol; 37.9 μg) to yield a 5 mM complex solution, which was equilibrated at room temperature for one hour in an Eppendorf™ LoBind Microcentrifuge tube (1.5 mL). Then, TRIS buffer (100 mM; pH 8) was added to yield a final metal-complex concentration of 100 μM and final buffer concentration of 10 mM. The solution equilibrated at room temperature for one hour and then the pH was confirmed to be approximately pH 8 with pH paper. An aliquot of this solution (10 μL) was analyzed by UPLC-MS and absorption spectroscopy. The corresponding data are provided in Supplementary Figures 12–13, which support the formation of the $[La^{III}(HOPO)]^{1-}$ complex under these conditions, which were mirrored in subsequent actinium studies. Furthermore, the $[La^{III}(HOPO)]^{1-}$ was formed at higher concentrations (50 μM) and monitored by UV-visible spectroscopy. The shift of the free HOPO peaks centered around 325 nm upon the addition of La^{III} is also indicative of metal complexation and formation of the $[La^{III}(HOPO)]^{1-}$ complex (Supplementary Figure 14).[18]

Characterization of Ac^{III}–HOPO–Scn crystals over time.

The crystals used for protein crystallographic measurements were removed from their CryoLoops™, dissolved into HCl (0.1M, 100 μL), and transferred to an Eppendorf tube (1.5 mL). The radioactivity of this sample was routinely measured using γ-spectrometry over the course of one year. The ingrowth of ^{227}Ac daughter isotopes, namely ^{227}Th, was monitored over time using the γ-line at 235.96 keV. The experimental data are plotted and compared to theoretical calculations. Importantly, the experimental activity of ^{227}Th never approaches zero, indicating that ^{227}Ac must be present in the sample (and thus the crystals measured). If this was not the case, the activity of ^{227}Th would approach zero, as is observed in the calculated decay curve. Rather, secular equilibrium appears to be reached after approximately 112 days, as indicated by the plateauing of the activity of ^{227}Th beyond this point. The calculated ^{227}Ac and ^{227}Th activities were modelled by the radioactive decay equation, $A = A_0 e^{-\lambda t}$ (A = activity; A_0= initial activity, λ =decay constant; t=time elapsed). A_0 for ^{227}Th was based on the first experimental ^{227}Th γ-measurement. A_0 for ^{227}Ac was the average of the ^{227}Th experimental activities after secular equilibrium was reached, at which point the activity of ^{227}Th should equal the activity of ^{227}Ac. The calculated versus experimental data are reported in Supplementary Figure 17.

Moreover, an LSC measurement of this crystal solution further confirmed the presence of ^{227}Ac directly, collected 1.5 years after the crystals were grown (Supplementary Figure 18).

Cumulative stability constants.

The cumulative stability constant ($\beta'_{1,2}$ value) for $^{227}Ac^{III}$ with HOPO and siderocalin can be determined by multiplying together the respective equilibrium constants as outlined in the following equations. The values for K_1 (formation constant of $[Ac^{III}(HOPO)]^{1-}$) and K_2 (inverse of dissociation constant of Ac^{III}–HOPO–Scn) were determined separately via Supplementary Equations 1–2 and Supplementary Equations 3–4, respectively.

$$\mathrm{M + L + Scn} \leftrightarrow \mathrm{[Scn{:}\,M{:}\,L]} \qquad \text{(Supplementary Equation 5)}$$

$$\beta'_{1,2} = \frac{\mathrm{[Scn{:}M{:}L]}}{\mathrm{[Scn][M][L]}} = \frac{\mathrm{[ML]}}{\mathrm{[M][L]}} \times \frac{\mathrm{[Scn{:}ML]}}{\mathrm{[Scn][ML]}} = K_1 K_2 \qquad \text{(Supplementary Equation 6)}$$

3. Supplementary Figures

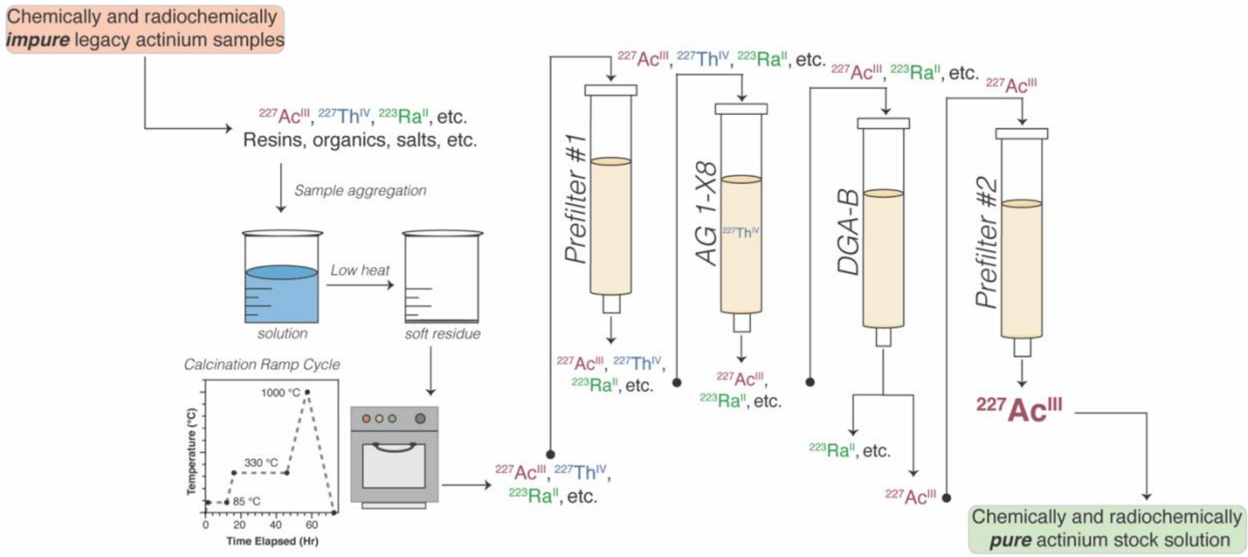

Supplementary Figure 1 | Visualization of the established methods used in this work to chemically and radiochemically purify ^{227}Ac.[5, 6, 7, 8]

Chemically and radiochemically pure actinium stock solutions were obtained from legacy actinium samples after consolidation, calcination, reconstitution, and elution through ion exchange columns.

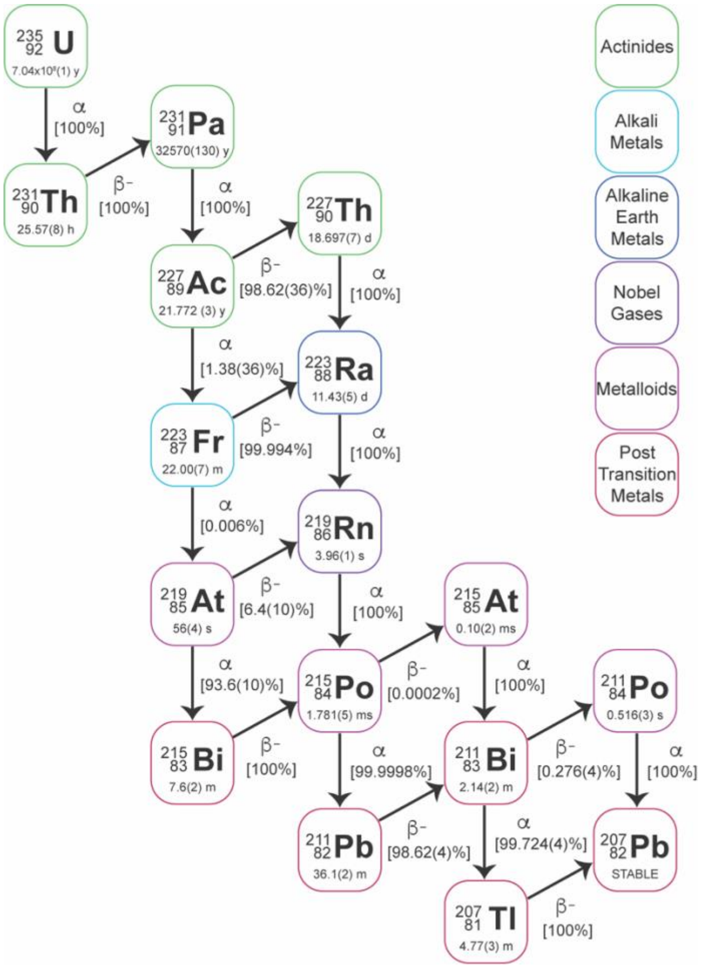

Supplementary Figure 2 | Decay Scheme of Uranium-Actinium Series that includes ^{227}Ac.

The radioactive decay scheme of ^{227}Ac. As part of the uranium-actinium series (or 4*n* + 3 series), ^{227}Ac decays to stable lead-207 (^{207}Pb) through many decay products. Note the differences in chemical identities and half-lives throughout the decay scheme, which highlight the nuances of both purifying ^{227}Ac and performing ^{227}Ac chemistry. Decay chain information was compiled from the National Nuclear Data Center (NNDC).[30]

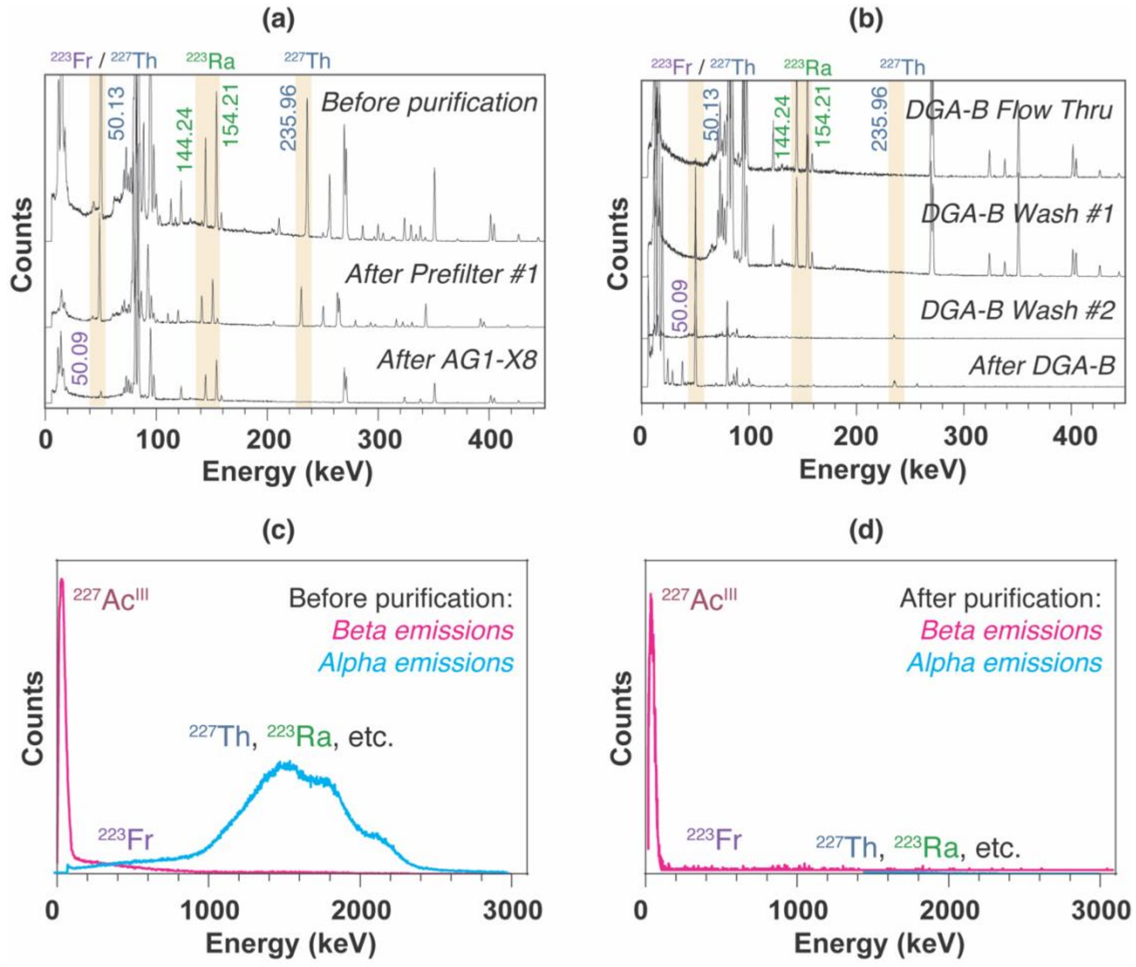

Supplementary Figure 3 | Representative LSC and γ spectroscopic measurements collected throughout the ^{227}Ac reprocessing procedure. Radiochemical purity was assessed directly with liquid scintillation counting (LSC) through actinium β emission and indirectly with γ spectroscopy through daughter ingrowth.

(a) γ-spectra of the ^{227}Ac stock solution before purification (top), after prefilter #1 (middle), and after anion exchange and removal of ^{227}Th^{IV} (bottom). Regions of interest are highlighted (tan), and notable ^{227}Th^{IV} (blue), ^{223}Fr^{I} (purple), and ^{223}Ra^{II} (green) γ-emissions are labelled accordingly.

(b) γ-spectra of the ^{227}Ac stock solution after passing through DGA-B resin to separate the ^{223}Ra^{II} (top), subsequent washes of the DGA-B resin (middle spectra), and elution off the DGA-B resin yield the purified stock of ^{227}Ac^{III}. Not shown is the spectrum after the final column (prefilter #2), albeit similar to the DGA-B elution profile.

(c) Alpha (blue) and beta (pink) emissions measured by LSC of the ^{227}Ac stock before purification. Approximate regions wherein relevant isotopes will decay, including ^{227}Th^{IV} (blue), ^{223}Fr^{I} (purple), and ^{223}Ra^{II} (green), are noted.

(d) Alpha (blue) and beta (pink) emissions measured by LSC of the ^{227}Ac stock after purification. Approximate regions wherein relevant isotopes will decay, including ^{227}Th^{IV} (blue), ^{223}Fr^{I} (purple), and ^{223}Ra^{II} (green), are noted.

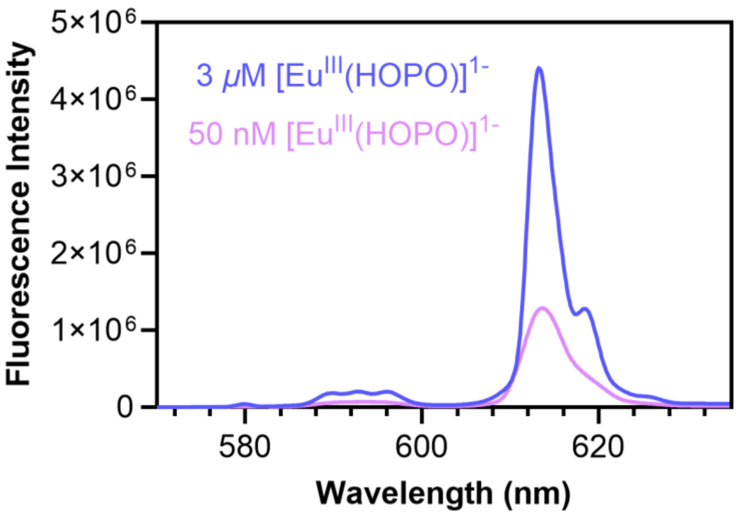

Supplementary Figure 4 | Fluorescence of [Eu^{III}(HOPO)]$^{1-}$ as a function of concentration and slit width.

Due to the need to minimize the amount of material in fluorescence competition titration experiments, the starting concentration of [Eu^{III}(HOPO)]$^{1-}$ was scaled down considerably. As a result, the resolution of the Eu^{III} *f*-*f* transitions were impacted when comparing solutions of 3 μM [Eu^{III}(HOPO)]$^{1-}$ (blue trace; slit size = 2 mm) vs. 50 nM [Eu^{III}(HOPO)]$^{1-}$ (purple trace; slit size = 4 mm), both collected in 0.1 M TRIS (pH 7.36) with the ionic strength of the solution maintained at 0.5 M with $KCl_{(aq)}$. Despite the loss of resolution, the data can still be reliably fitted to determine the conditional stability constants for Ac^{III}.

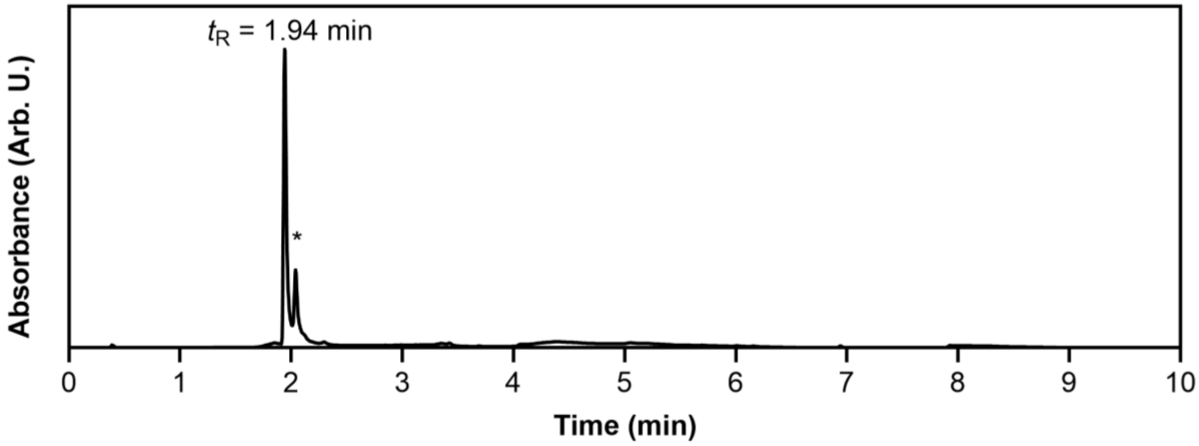

Supplementary Figure 5 | UPLC chromatograph of [Eu^{III}(HOPO)]$^{1-}$ monitored at 300 nm.

The asterisk indicates the peak corresponding to the Fe^{III} complex, which formed in the UPLC-MS system.

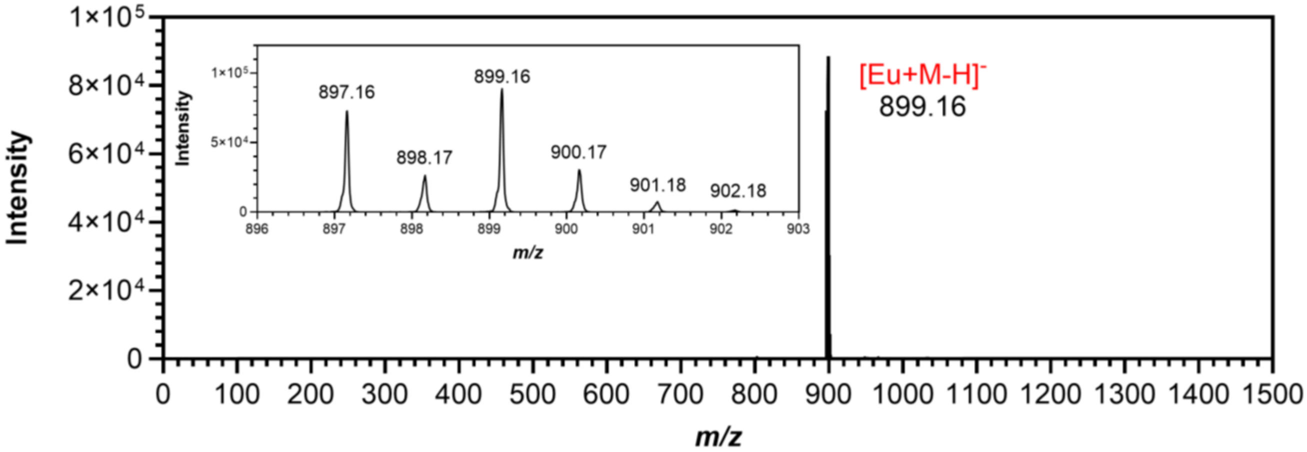

Supplementary Figure 6 | QTOF-MS spectrum of [Eu^{III}(HOPO)]$^{1–}$.

m/z = 899.16 ([Eu+M–H]$^{–}$, M = HOPO, Calc = 889.15 for $C_{34}H_{34}EuN_8O_{12}$). Inset shows the isotopic distribution.

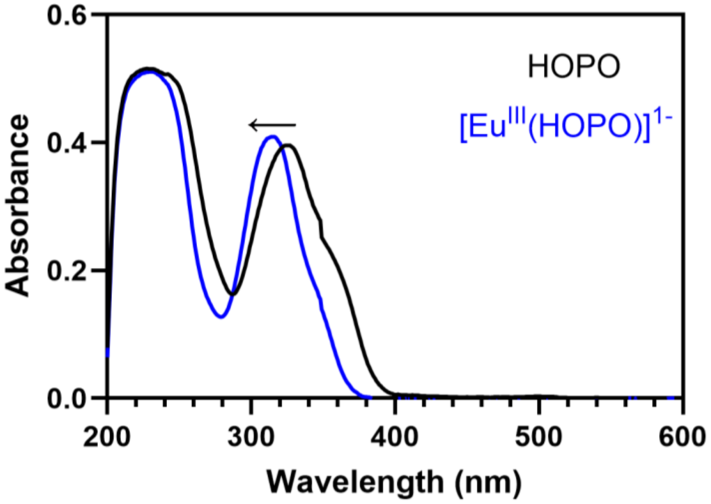

Supplementary Figure 7 | Absorption spectrum of HOPO in the absence and presence of Eu^{III} ([HOPO] = [Eu^{III}] = 5.0 × 10-6 M, *I* = 0.5 M KCl, T = 25 °C).

The absorption bands associated with HOPO undergo a blue shift (indicated by the arrow) in the presence of europium, which is consistent with metal complexation under these buffered conditions (pH 7.36).[17]

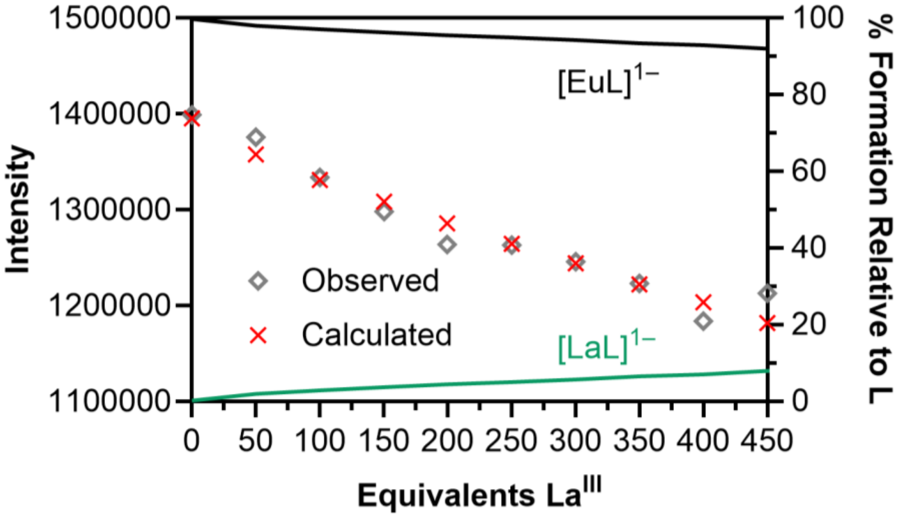

Supplementary Figure 8 | Data fitting and species distribution of [Eu^{III}(HOPO)]$^{1-}$ upon titration with La^{III}.

Fluorescence changes (λ_{ex} = 325 nm, λ_{em} = 570 nm–630 nm) of a solution containing HOPO (L) and Eu^{III} ([L] = [M] = 1 × 10^{-8} M) upon titration with La^{III} (T = 25 °C, I = 0.5 M KCl, pH = 7.36).

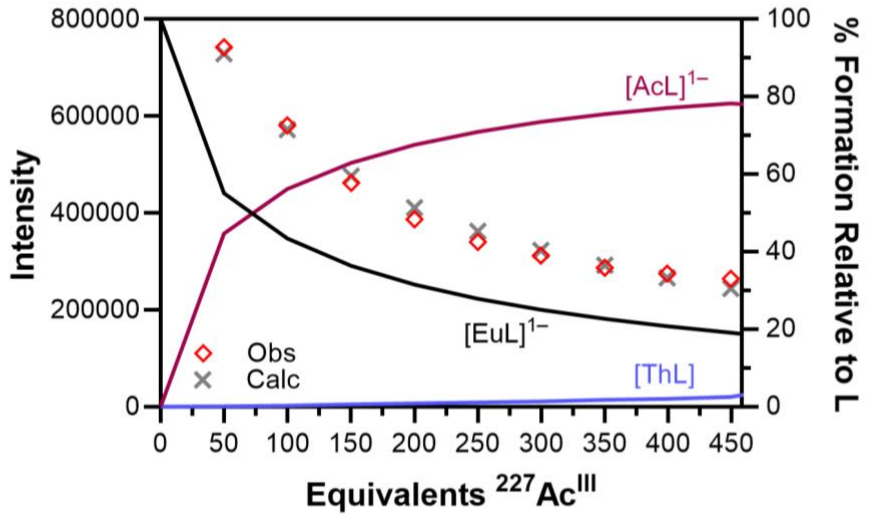

Supplementary Figure 9 | Data fitting and species distribution of [Eu^{III}(HOPO)]$^{1-}$ upon titration with Ac^{III}.

Fluorescence changes (λ_{ex} = 325 nm, λ_{em} = 570 nm–630 nm) of a solution containing HOPO (L) and Eu^{III} ([L] = [M] = 1 × 10^{-8} M) upon titration with Ac^{III} (T = 25 °C, I = 0.5 M KCl, pH = 7.36). The contribution of [Th^{IV}(HOPO)] was included in the fit as ^{227}Th is the first decay product of ^{227}Ac, for which the concentration was determined at each titration point through γ spectroscopy.

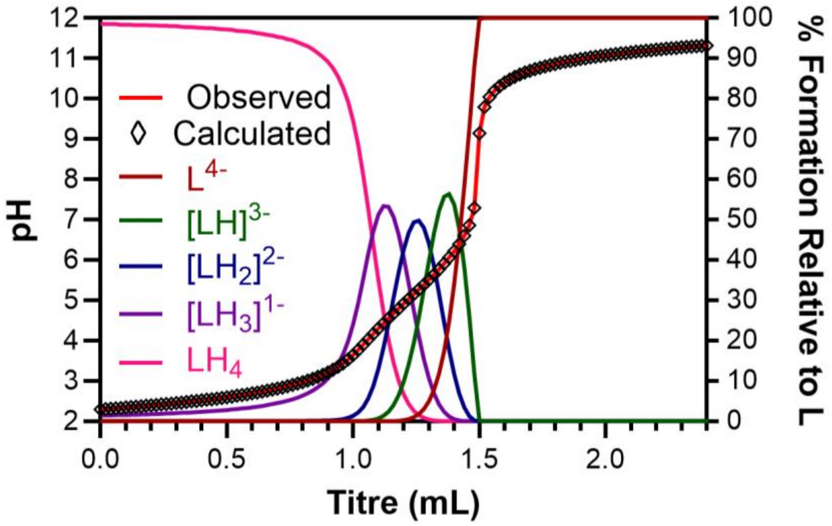

Supplementary Figure 10 | Potentiometric titration and ligand speciation.

HOPO speciation over Representative potentiometric titration curve of HOPO with respective data fitting and species distribution over the titration pH range ([L] = 5.96 × 10-4 M, *I* = 0.5 M KCl, T = 25 °C).

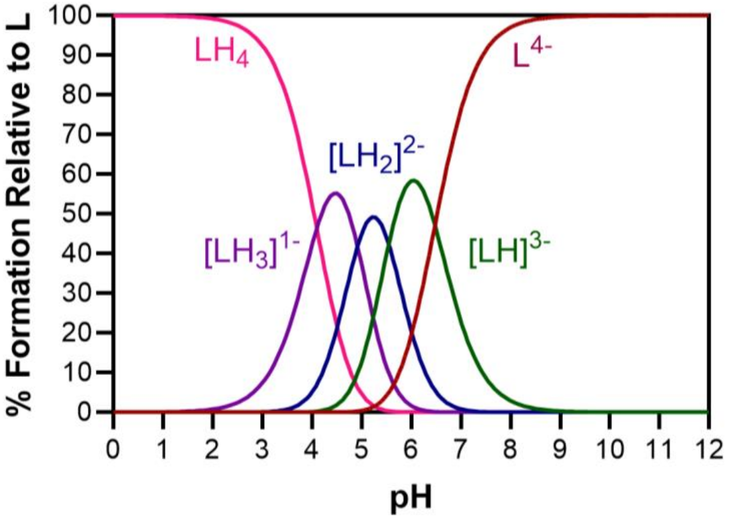

Supplementary Figure 11 | HOPO ligand speciation.

Species distribution diagram for HOPO (L) from pH 0 – 12 (*I* = 0.5 M KCl, T = 25 °C).

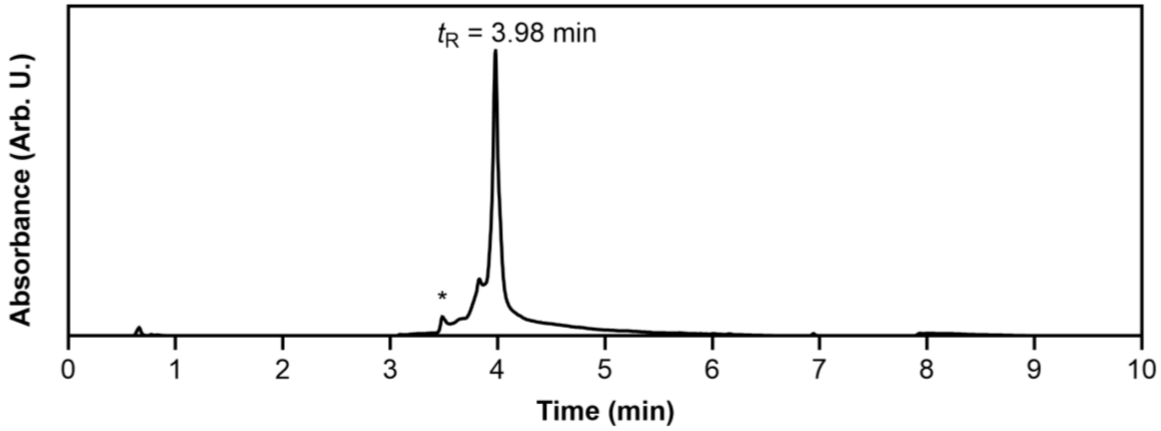

Supplementary Figure 12 | UPLC chromatograph of $[La^{III}(HOPO)]^{1-}$ monitored at 300 nm.
The asterisk indicates the peak corresponding to the Fe^{III} complex, which formed in the UPLC-MS system (see Supplementary Figure 13).

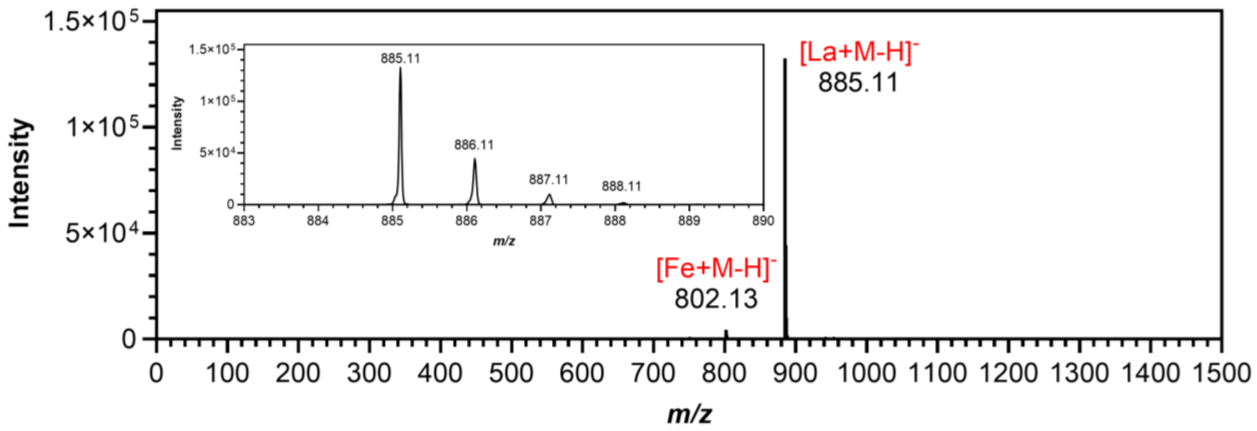

Supplementary Figure 13 | QTOF-MS spectrum of $[La^{III}(HOPO)]^{1-}$.
m/z = 885.11 ($[La+M-H]^-$, M = HOPO, Calc = 885.14 for for $C_{34}H_{34}LaN_8O_{12}$), 802.13 ($[Fe+M-H]^-$, M = HOPO, Calc = 802.16 for $C_{34}H_{34}FeN_8O_{12}$). Inset shows the isotopic distribution.

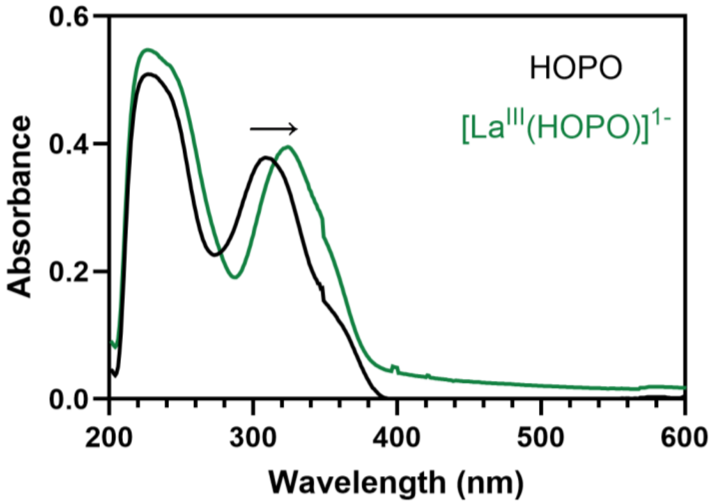

Supplementary Figure 14 | Absorption spectrum of HOPO in the absence and presence of La^{III} ([HOPO] = [La^{III}] = 5.0 × 10-6 M, *I* = 0.5 M KCl, T = 25 °C).

The absorption bands associated with HOPO undergo a red shift (indicated by the arrow) in the presence of lanthanum, which is consistent with metal complexation under these buffered conditions (pH 7.36).[17]

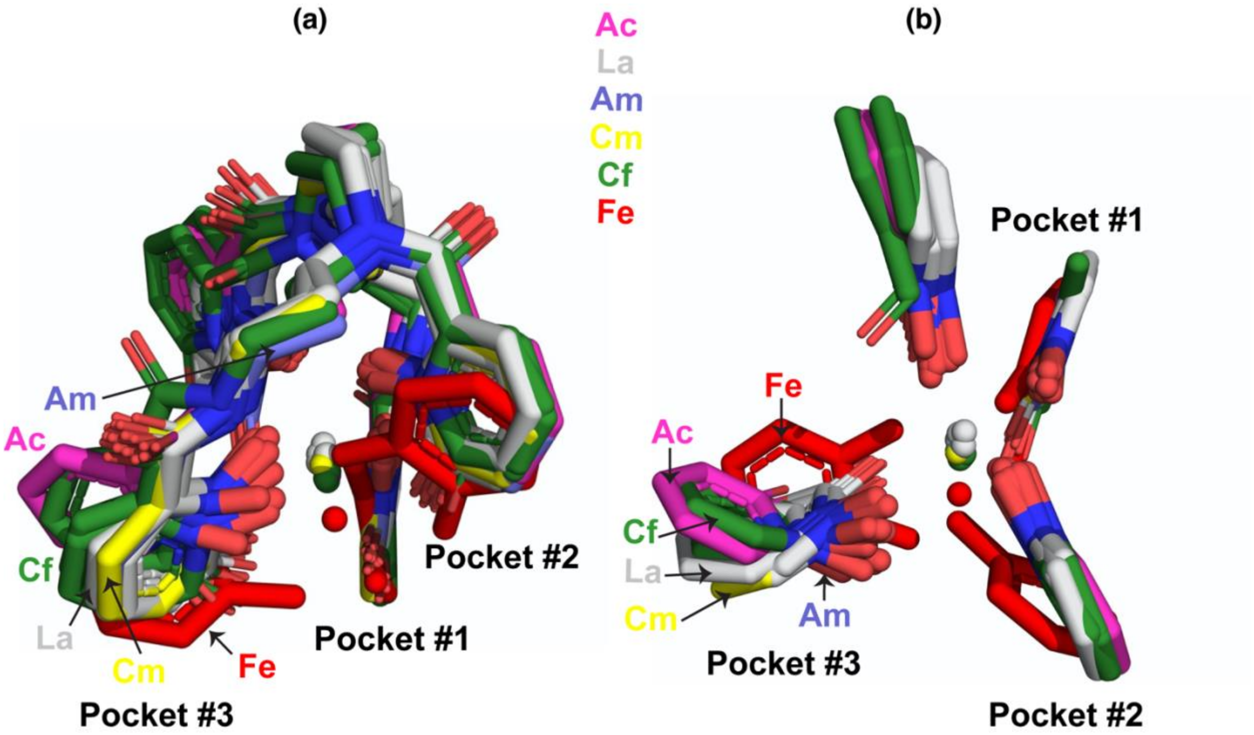

Supplementary Figure 15 | Stereoviews of M^{III}–HOPO structures in the calyx of Scn.

Pockets that house the ligand aryl groups are labelled. The ball-and-stick representations of M^{III}–HOPO molecules from crystal structures [La (grey) and Ac (magenta) structures described in this work, plus PDB accession codes 4ZHG (Am–HOPO; blue), 4ZHF (Cm–HOPO; yellow), 5KIC (Cf–HOPO), and 1L6M (Fe–Ent; red)] are superimposed.[2, 20, 31]

(a) The ball-and-stick representations of M^{III}–HOPO molecules, illustrating the entirety of the linear spermine backbones and HOPO binding groups (or catecholamide [CAM] binding groups for Fe^{III}–Ent).

(b) The ball-and-stick representations of M^{III}–HOPO molecules, rotated 180 degrees from (A), and illustrating only the HOPO binding groups (or CAM groups for Fe^{III}–Ent) for clarity.

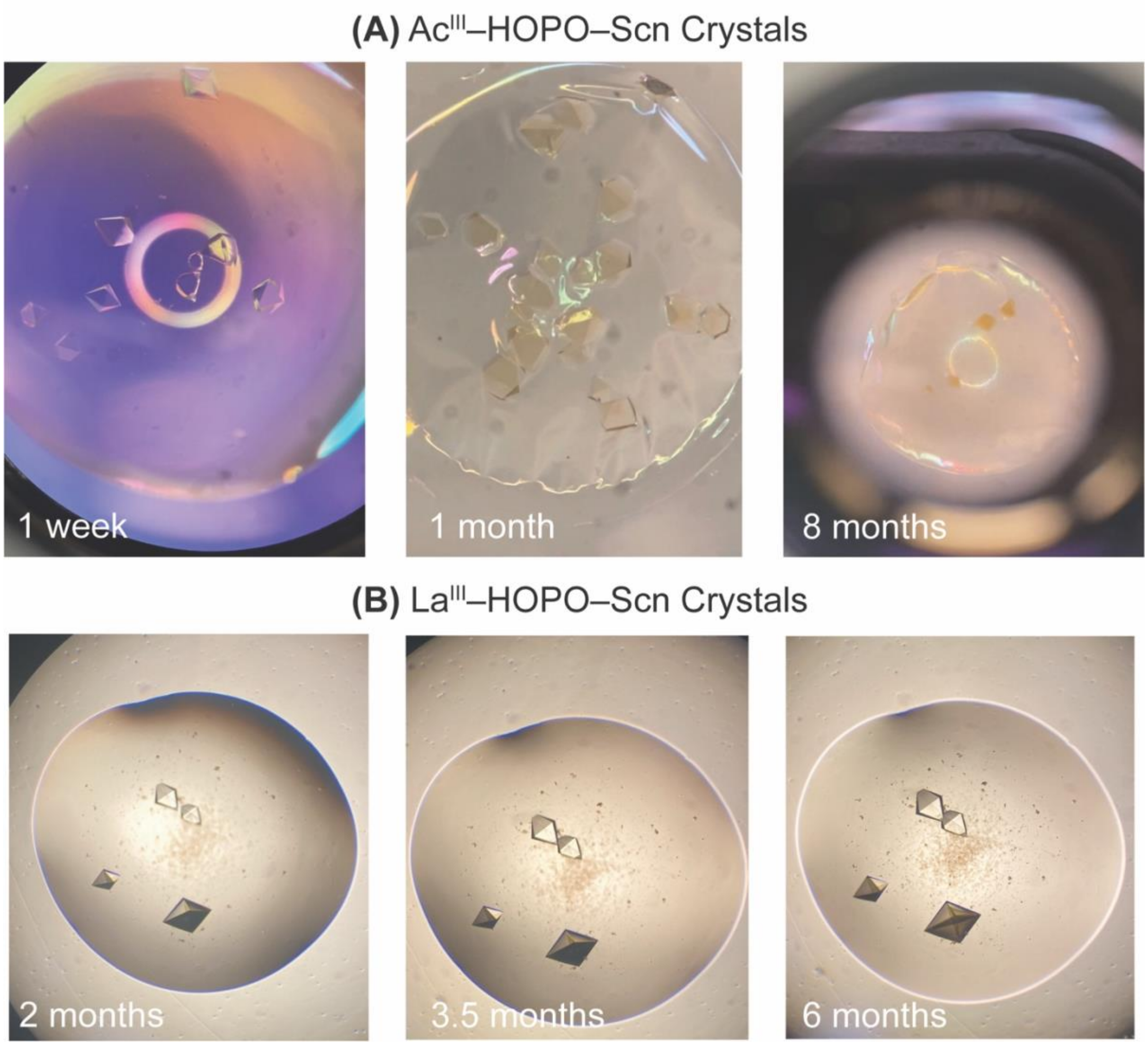

Supplementary Figure 16 | Optical images of Ac^{III}–HOPO–Scn and La^{III}–HOPO–Scn crystals over time.

(a) Ac^{III}–HOPO–Scn crystals and (b) La^{III}-HOPO-Scn crystals under an optical microscope as a function of time (t=0 d when crystallization solutions were plated on crystallization plates). As both systems crystallize in the same space group and are colorless initially, the crystal color changes in (A) indicate radiation damage to crystals, which is absent in the non-radioactive lanthanum images in (B). Note the purple hue in (A)-1 week is not due to the crystals (they are colorless), but instead due to the microscope stage. This purple hue is absent in (A) at 1 and 8 months because a white cloth was placed below the sample to highlight the change in crystal colors. The images in (B) were taken on a different optical microscope that did not have the same background. The approximate size of the crystals ranged from 0.05 mm x 0.05 mm to 0.1 mm x 0.2 mm.

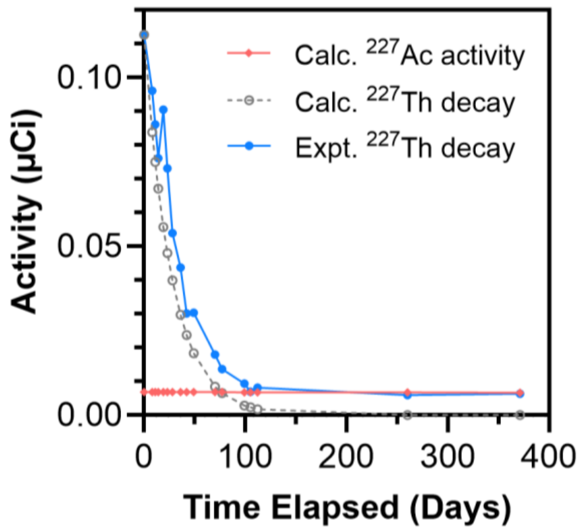

Supplementary Figure 17 | Actinium quantification in protein crystals.

γ-spectroscopic measurements of Ac^{III}-HOPO-Scn crystals dissolved in 0.1 M $HCl_{(aq)}$, which tracked the ^{227}Th activity as a function of time (blue trace) compared to the calculated decays of ^{227}Th (gray trace) and ^{227}Ac (salmon trace).

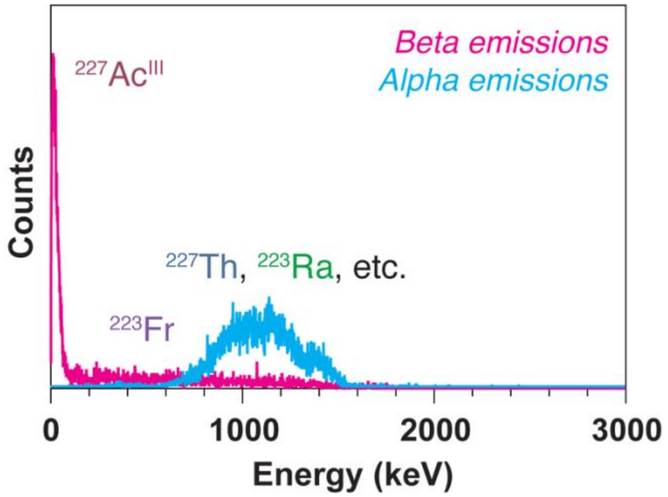

Supplementary Figure 18 | LSC spectrum of Ac^{III}-HOPO-Scn crystals dissolved in 0.1 M $HCl_{(aq)}$ measured twenty-one months after initial crystallization.

Alpha (blue) and beta (pink) emissions measured by LSC on an aliquot (1 μL) of ^{227}Ac–HOPO–Scn crystals dissolved in 0.1 M $HCl_{(aq)}$. Approximate regions wherein relevant isotopes will decay, including $^{227}Th^{IV}$ (blue), $^{223}Fr^{I}$ (purple), and $^{223}Ra^{II}$ (green), are noted.

(a)

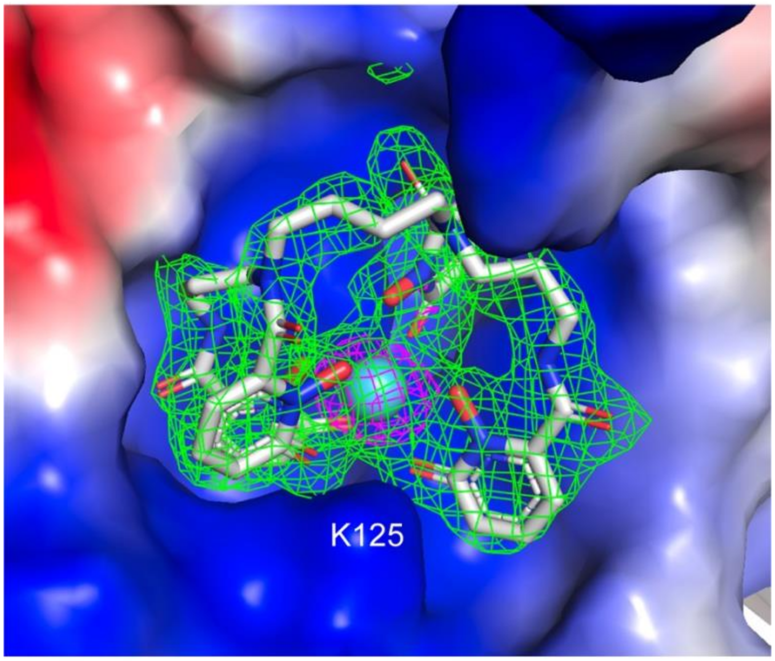

(b)

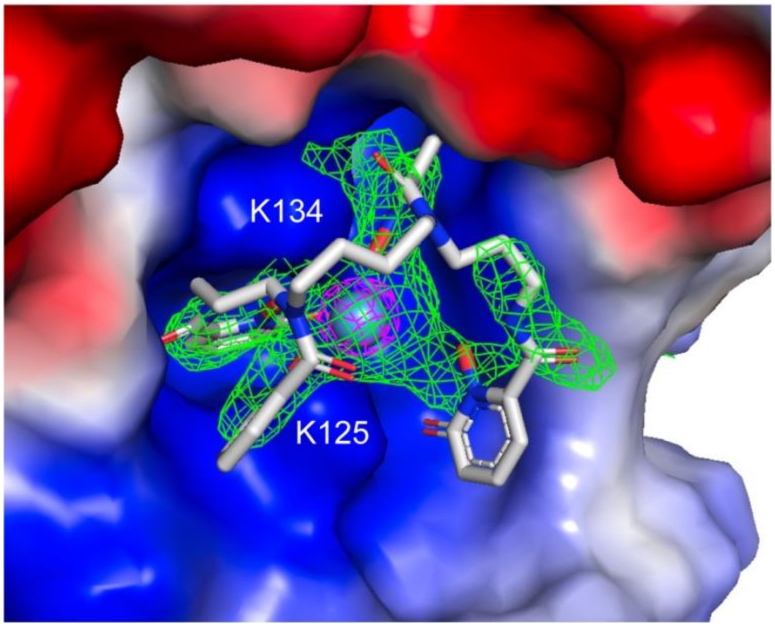

Supplementary Figure 19 | OMIT difference maps for the structures presented herein.

(a) The OMIT difference map for the La^{III}–HOPO–Scn, contoured at 3σ (green) and 10σ (magenta). Scn is represented as a molecular surface colored by electrostatic potential.

(b) The OMIT difference map for the Ac^{III}–HOPO–Scn, contoured at 2σ (green) and 10σ (magenta). Scn is represented as a molecular surface colored by electrostatic potential.

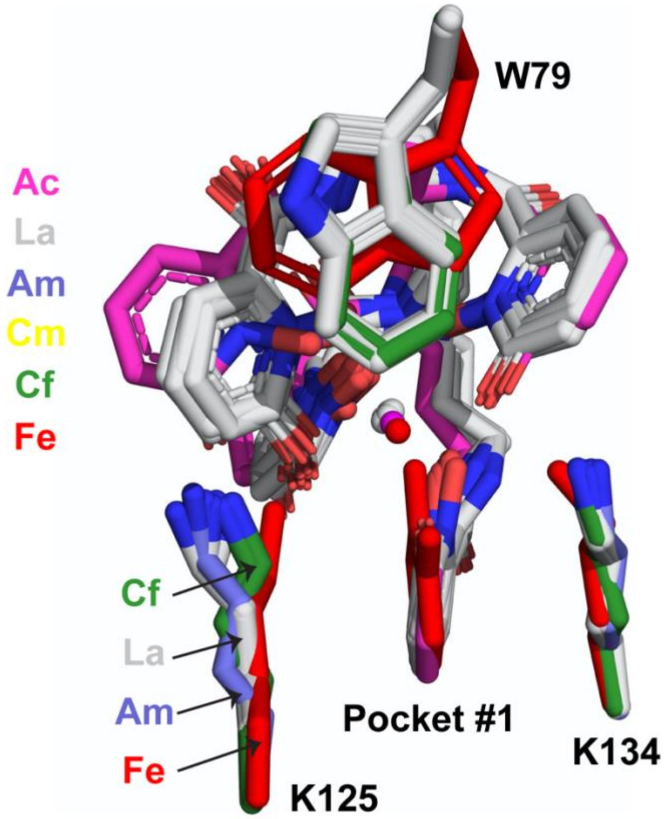

Supplementary Figure 20 |A stereoview of the side chains of key calyx amino acid residues during M^{III}–HOPO recognition.

Key positions discussed in the text are numbered, including tryptophan (W79) and lysine (K125, K134) residues. The ball-and-stick representations of M^{III}–HOPO molecules from crystal structures [La (grey) and Ac (magenta) structures described in this work, plus PDB accession codes 4ZHG (Am–HOPO; blue), 4ZHF (Cm–HOPO; yellow), 5KIC (Cf–HOPO), and 1L6M (Fe–Ent)] are superimposed.[2, 20, 31]

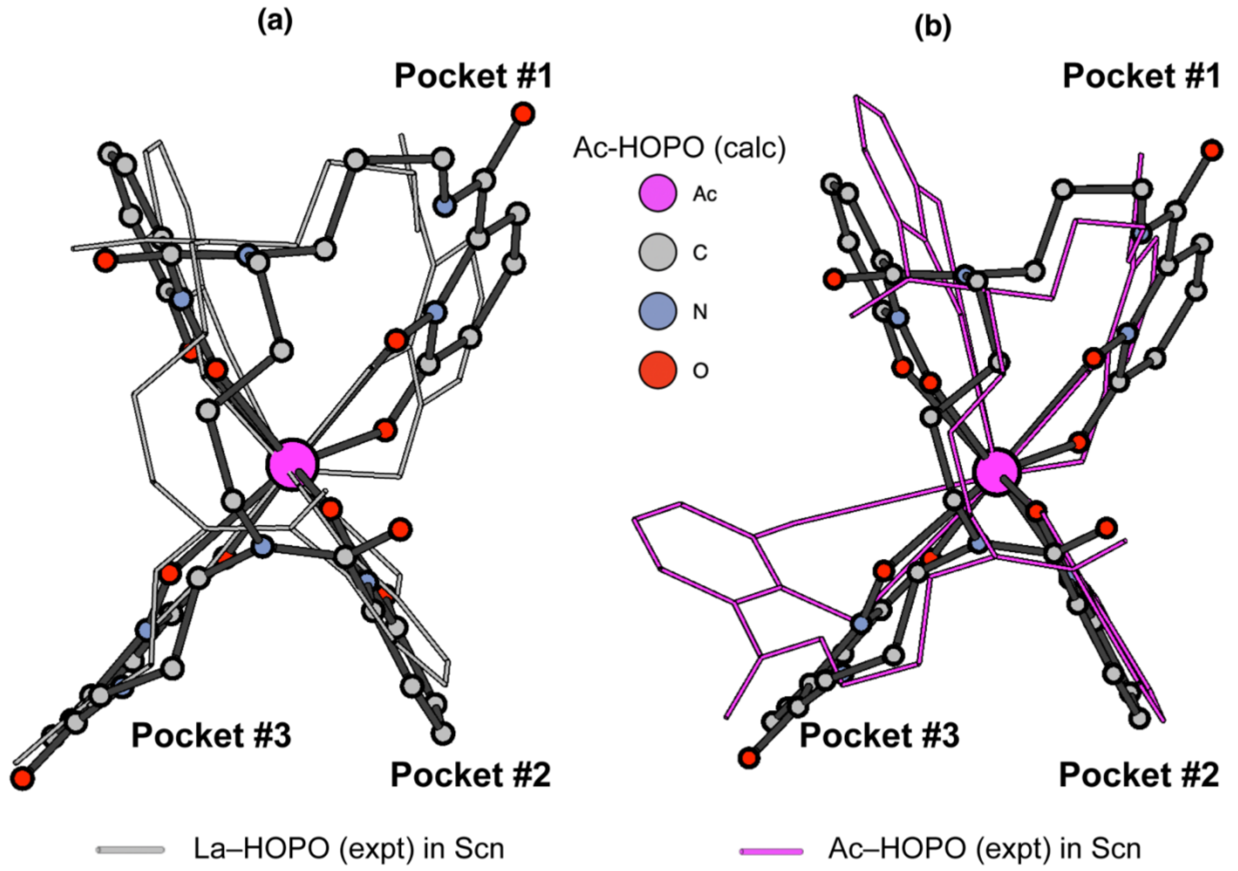

Supplementary Figure 21 | Superimposed structures of M^{III}–HOPO in the calyx of Scn overlayed with the calculated structure of $[Ac^{III}(HOPO)]^{1-}$.

Siderocalin pockets that house the ligand aryl groups are labelled. The wire frame representations of M^{III}–HOPO molecules crystallized within siderocalin [La (grey) and Ac (magenta)] described in this work are superimposed with the ball-and-stick representation of the $[Ac^{III}(HOPO)]^{1-}$ structure calculated previously with DFT (PBE functional, relativistic ZORA Hamiltonian, and triple-ζ plus two polarization function (TZ2P) basis sets).[32]

(a) The wire frame representation of the La^{III}–HOPO molecule when bound to Scn overlayed with the calculated $[Ac^{III}(HOPO)]^{1-}$ ball-and-stick structure.

(b) The wire frame representation of the Ac^{III}–HOPO molecule when bound to Scn overlayed with the calculated $[Ac^{III}(HOPO)]^{1-}$ ball-and-stick structure.

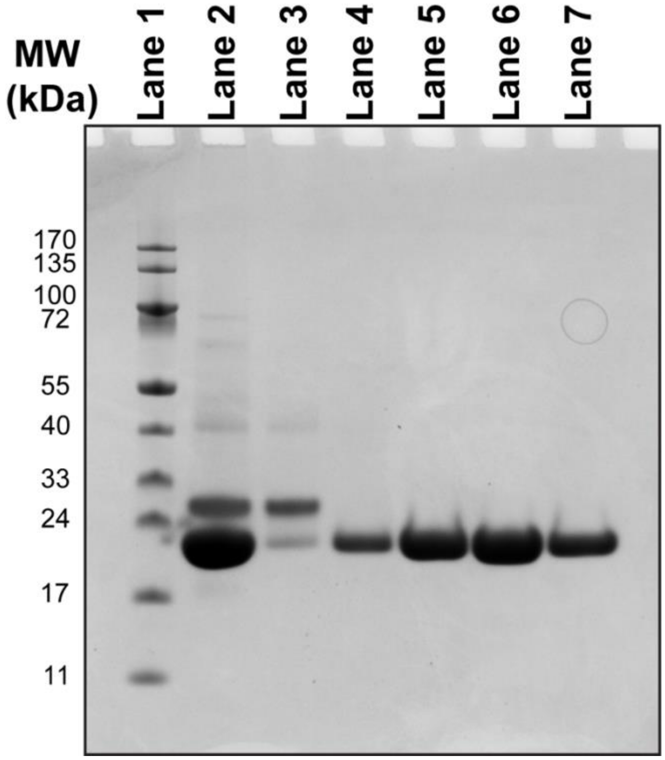

Supplementary Figure 22 | SDS-PAGE gel of siderocalin purification.

Lane 1: Fisher BioReagents™ EZ-Run™ Protein Ladder with corresponding molecular weights (kDa) noted on the left side of the image. Lane 2: Scn load before size exclusion column chromatography. Lane 3: First elution peak after size exclusion column chromatography; Scn containing glutathione *S*-transferase (GST) tag. Lane 4 – Lane 7: Fractions of the second elution peak after size exclusion column chromatography; purified Scn used in these studies, with a molecular weight of approximately 20.5 kDa.

4. Supplementary Tables

Supplementary Table 1 | Tabulation of $[La^{III}(HOPO)]^{1-}$ conditional thermodynamic constants (log β'_{ML}) determined from competition titrations with $[Eu^{III}(HOPO)]^{1-}$ under various conditions.[a]

Investigated Condition	Tested Conditions	log β'_{ML} Value	Reference Conditions	log β'_{ML} Value
Buffer	$[Eu^{III}]$ = [HOPO] = 3 μM<br>I = 0.1 M KCl<br>0.1 M Tris, pH 7.36	18.9	$[Eu^{III}]$ = [HOPO] = 3 μM<br>I = 0.1 M KCl<br>0.1 M HEPES, pH 7.4	16.4(3)[b]
Semi-Batch Titration	$[Eu^{III}]$ = [HOPO] = 3 μM<br>I = 0.5 M KCl<br>0.1 M Tris, pH 7.36<br>Semi-batch titration	17.08(4)	$[Eu^{III}]$ = [HOPO] = 3 μM<br>I = 0.5 M KCl<br>0.1 M Tris, pH 7.36<br>Batch titration	17.3
Equilibration Time	$[Eu^{III}]$ = [HOPO] = 50 nM<br>I = 0.5 M KCl<br>0.1 M Tris, pH 7.36<br>1-hour equilibration	17.5	$[Eu^{III}]$ = [HOPO] = 50 nM<br>I = 0.5 M KCl<br>0.1 M Tris, pH 7.36<br>2-day equilibration	17.1
M–L Concentration	$[Eu^{III}]$ = [HOPO] = 50 nM<br>I = 0.5 M KCl<br>0.1 M Tris, pH 7.36	17.2(1)	$[Eu^{III}]$ = [HOPO] = 10 nM<br>I = 0.5 M KCl<br>0.1 M Tris, pH 7.36	16.50(3)

[a] If no uncertainty provided, n = 1 for each tested condition. If uncertainty provided (n = 3), the uncertainty corresponds to the standard deviation of the last digit.
[b] Supplementary Reference 17.

Supplementary Table 2 | Protonation constants of HOPO used in the refinement of the conditional stability constants reported herein.[a]

Parameter	*pKa value*
pK_{a1}	6.48(10)
pK_{a2}	5.55(1)
pK_{a3}	4.92(3)
pK_{a4}	4.10(3)
$\sum pK_a$	21.05

[a] T = 25 °C, I = 0.5 M KCl. The uncertainty corresponds to the standard deviation of the last digit of the protonation constant (n = 3).

Supplementary Table 3 | Metal hydrolysis constants used in the refinement of the conditional stability constants reported herein.

	**La^{III}**	
Species	*log β*	*Reference*
$[La(OH)]^{2+}$	–8.8	
$La(OH)_3$	–20.3	17
$[La_2(OH)_2]^{4+}$	–17.1	
	**Eu^{III}**	
Species	*log β*	*Reference*
$[Eu(OH)]^{2+}$	–8.1	
$[Eu(OH)_2]^{1+}$	–14.4	17
$Eu(OH)_3$	–24.5	
	**Th^{IV}**	
Species	*log β*	*Reference*
$[ThOH]^{3+}$	–2.5	
$[Th(OH)_2]^{2+}$	–6.2	
$Th(OH)_4$	–17.4	
$[Th_2(OH)_2]^{6+}$	–5.9	
$[Th_2(OH)_3]^{5+}$	–6.8	19
$[Th_4(OH)_8]^{8+}$	–20.4	
$[Th_4(OH)_{12}]^{4+}$	–26.6	
$[Th_6(OH)_{14}]^{10+}$	–36.8	
$[Th_6(OH)_{15}]^{9+}$	–38.06	
	**Ac^{III}**	
Species	*log β*	*Reference*
$[Ac(OH)]^{2+}$	–9.4	33

Supplementary Table 4 | Tabulation of *DynaFit* parameters used to determine the average dissociation constant (K_D) of $[Ac^{III}(HOPO)]^{1-}$ and siderocalin (Scn).

Parameter Description	*Trial 1*	*Trial 2*	*Trial 3*
K_D (nM)	6.60	5.74	3.91
[Scn] (nM)	134.90	100.60	97.50
r(Scn)	0.0076	0.0100	0.0102
r(Scn.AcHOPO)	0.0011	0.0018	0.0025

Supplementary Table 5 | Tabulation of *DynaFit* parameters used to determine the average dissociation constant (K_D) of HOPO and siderocalin (Scn).

Parameter Description	*Trial 1*	*Trial 2*	*Trial 3*
K_D (nM)	66.00	24.80	36.80
[Scn] (nM)	0.14	124.40	0.10
r(Scn)	7.1000	0.0085	9.6000
r(Scn.HOPO)	1.3000	0.0006	0.9300

Supplementary Table 6 | Tabulation of *DynaFit* parameters used to determine the average dissociation constant (K_D) of $[La^{III}(HOPO)]^{1-}$ and siderocalin (Scn).

Parameter Description	*Trial 1*	*Trial 2*	*Trial 3*
K_D (nM)	21.20	14.20	25.30
[Scn] (nM)	75.00	78.70	50.70
r(Scn)	0.0134	0.0128	0.0202
r(Scn.LaHOPO)	0.0026	0.0022	0.0040

Supplementary Table 7 | Macromolecular crystallographic data collection and refinement statistics for Ac^{III}–HOPO–Scn and La^{III}–HOPO–Scn structures.[a]

	Ac^{III}–HOPO–Scn	La^{III}–HOPO–Scn
Data Collection Parameters		
Space Group	$P4_12_12$	$P4_12_12$
Cell Dimensions (Å)	114.8, 114.8, 119.4	116.1, 116.1, 117.6
Resolution Range (Å)	82.73-2.04 (2.10-2.04)	50.00-2.00 (2.03-2.00)
Wavelength (Å)	1.000	1.031
Unique Reflections	48793(3829)	55159(2710)
Completeness (%)	96.2(97.8)	100.0(100.0)
Average Redundancy	6.2(5.9)	11.5(11.9)
R_{merge} (%)	9.1(47.3)	8.0(48.3)
$I/\sigma(I)$	8.5(2.3)	41.7(5.1)
Structural Refinement Parameters		
Resolution (Å)	50.00-2.08	50.00-2.00
No. of Reflections		
all/test	45773/2438	52322/2714
No. of non-hydrogen atoms [average B-factor (Å^2)]		
Protein	4123(73)	4193(36)
Metal(III)–HOPO	82(100)	165(47)
Others (solvent)	42(63)	191(41)
R_{cryst}/R_{free}	22.5/25.5	20.4/22.7
Rmsd		
Bonds (Å)/Angles (°)	0.016/1.435	0.008/1.493
Estimate of Coordinate Error (Å)		
Maximum likelihood e.s.u	0.117	0.088
Ramachandran values (*ProCheck*)		
Favored region (%)	97.0	97.8
Allowed region (%)	3.0	2.2
Outlier region (%)	0	0
Molprobity percentile (score)	99(1.39)	100(1.19)

[a] Values in parentheses are for reflections in the highest resolution shell.

Supplementary Table 8 | Tabulation of Ac^{III}–HOPO interatomic distances in the solid-state structure of Ac^{III}–HOPO–Scn.

Ac–O Bond Type	*Chain A Ac–O (Å)*[a]	*Chain B Ac–O (Å)*[a]	*Chain C Ac–O (Å)*	*$Ac–O_{avg}$ (Å)*[b]
N-Oxide	2.72	2.26	2.40	2.9(5)
N-Oxide	-	-	2.77	
N-Oxide	-	-	3.36[c]	
N-Oxide	-	-	3.60[c]	
Ketone	3.14	3.22	2.70	3.5(8)
Ketone	-	-	2.87	
Ketone	-	-	4.38[c]	
Ketone	-	-	4.87[c]	
$Ac–O_{avg}$ (Å)[b]	2.9(2)	2.7(5)	3.4(8)	3.2(7)

[a] If no distance is provided, the binding group was not crystallographically refined.
[b] The uncertainty corresponds to the standard deviation of the last digit. Refer to the estimate of coordination error in Supplementary Table 7.
[c] Corresponds to aryl groups displaced from the protein calyx in Pockets #1 and #3.

Supplementary Table 9 | Tabulation of La^{III}–HOPO interatomic distances in the solid-state structure of La^{III}–HOPO–Scn.

La–O Bond Type	*Chain A La–O (Å)*	*Chain B La–O (Å)*	*Chain C La–O (Å)*	*$La–O_{avg}$ (Å)*[a]
N-Oxide	2.29	2.42	2.12	2.5(5)
N-Oxide	2.67	2.43	2.35	
N-Oxide	2.67	2.50	2.36	
N-Oxide	2.68	2.55	2.58	
Ketone	2.60	2.44	2.38	2.6(5)
Ketone	2.65	2.66	2.58	
Ketone	2.67	2.68	2.61	
Ketone	2.73	2.77	2.63	
$La–O_{avg}$ (Å)[a]	2.6(1)	2.6(1)	2.5(2)	2.5(2)

[a] The uncertainty corresponds to the standard deviation of the last digit. Refer to the estimate of coordination error in Supplementary Table 7.

Supplementary Table 10 | Examples of distances in the solid-state structures between the La^{III}/Ac^{III}–HOPO complexes and amino acid residues in the siderocalin calyx. Stereoviews of these amino acid residues during M^{III}–HOPO recognition are provided in Supplementary Figure 20.

Interaction	*Chain A (Å)*	*Chain B (Å)*	*Chain C (Å)*	*Avg. Distance (Å)*[a]
		**Ac^{III}–HOPO–Scn**		
Ac···$N\zeta_{K125}$	4.07	4.45	3.38	4.0(4)
Ac···$N\zeta_{K134}$	3.88	4.05	3.81	3.9(1)
		**La^{III}–HOPO–Scn**		
La···$N\zeta_{K125}$	4.59	4.33	4.49	4.5(1)
La···$N\zeta_{K134}$	4.27	4.56	4.53	4.5(1)

[a] The uncertainty corresponds to the standard deviation of the last digit. Refer to the estimate of coordination error for each structure in Supplementary Table 7.

5. Supplementary References

1. Abergel RJ, *et al.* Biomimetic actinide chelators: an update on the preclinical development of the orally active hydroxypyridonate decorporation agents 3,4,3-LI(1,2-HOPO) and 5-LIO(Me-3,2-HOPO). *Health Phys* **99**, 401-407 (2010).

2. Goetz DH, Holmes MA, Borregaard N, Bluhm ME, Raymond KN, Strong RK. The neutrophil lipocalin NGAL is a bacteriostatic agent that interferes with siderophore-mediated iron acquisition. *Mol Cell* **10**, 1033-1043 (2002).

3. Pettersen EF, *et al.* UCSF ChimeraX: structure visualization for researchers, educators, and developers. *Prot Sci* **30**, 70-82 (2021).

4. Johnson W, Chan E, Walsh E, Morte C, Lee D. "*InterSpec* v. 1.0. 9." Sandia National Lab. Albuquerque, NM, United States (2021).

5. Zielinska B, Apostolidis C, Bruchertseifer F, Morgenstern A. An improved method for the production of Ac-225/Bi-213 from Th-229 for targeted alpha therapy. *Solvent Extr Ion Exch* **25**, 339-349 (2007).

6. Ferrier MG, *et al.* Spectroscopic and computational investigation of actinium coordination chemistry. *Nat Commun* **7**, 12312 (2016).

7. Ferrier MG, *et al.* Synthesis and characterization of the actinium aquo ion. *ACS Cent Sci* **3**, 176-185 (2017).

8. Stein BW, *et al.* Advancing chelation chemistry for actinium and other +3 *f*-elements, Am, Cm, and La. *J Am Chem Soc* **141**, 19404-19414 (2019).

9. Bateman H. The solution of a system of differential equations occurring in the theory of radioactive transformations. *Proc Cambridge Philos Soc* **15**, 423-427 (1910).

10. Kossert K, Bokeloh K, Dersch R, Nähle O. Activity determination of ^{227}Ac and ^{223}Ra by means of liquid scintillation counting and determination of nuclear decay data. *Appl Radiat Isot* **95**, 143-152 (2015).

11. Marouli M, *et al.* Measurement of absolute γ-ray emission probabilities in the decay of ^{227}Ac in equilibrium with its progeny. *Appl Radiat Isot* **144**, 34-46 (2019).

12. Tollefson AD, *et al.* Measurement of 2^{27}Ac impurity in ^{225}Ac using decay energy spectroscopy. *Appl Radiat Isot* **172**, 109693 (2021).

13. Gans P, O'Sullivan B. *GLEE*, a new computer program for glass electrode calibration. *Talanta* **51**, 33-37 (2000).

14. Sweeton FH, Mesmer RE, Baes CF. Acidity measurements at elevated temperatures. VII. Dissociation of water. *J Solution Chem* **3**, 191-214 (1974).

15. Rossotti F, Rossotti H. Potentiometric titrations using Gran plots: A textbook omission. *J Chem Educ* **42**, 375 (1965).

16. Gans P, Sabatini A, Vacca A. Investigation of equilibria in solution. Determination of equilibrium constants with the *HYPERQUAD* suite of programs. *Talanta* **43**, 1739-1753 (1996).

17. Sturzbecher-Hoehne M, *et al.* 3,4,3-LI(1,2-HOPO): In vitro formation of highly stable lanthanide complexes translates into efficacious *in vivo* europium decorporation. *Dalton Trans* **40**, 8340-8346 (2011).

18. Deblonde GJP, Sturzbecher-Hoehne M, Abergel RJ. Solution thermodynamic stability of complexes formed with the octadentate hydroxypyridinonate ligand 3,4,3-LI(1,2-HOPO): a critical feature for efficient chelation of lanthanide(IV) and actinide(IV) ions. *Inorg Chem* **52**, 8805-8811 (2013).

19. Pham TA, Xu J, Raymond KN. A macrocyclic chelator with unprecedented Th^{4+} affinity. *J Am Chem Soc* **136**, 9106-9115 (2014).

20. Allred BE, *et al.* Siderocalin-mediated recognition, sensitization, and cellular uptake of actinides. *Proc Natl Acad Sci USA* **112**, 10342-10347 (2015).

21. Kuzmič P. Program *DYNAFIT* for the analysis of enzyme kinetic data: application to HIV proteinase. *Anal Biochem* **237**, 260-273 (1996).

22. Murshudov GN, Vagin AA, Dodson EJ. Refinement of macromolecular structures by the maximum-likelihood method. *Acta Crystallogr D: Biol Crystallogr* **53**, 240-255 (1997).

23. Bailey S. The *CCP4* suite-programs for protein crystallography. *Acta Crystallogr D: Struct Biol* **50**, 760-763 (1994).

24. Emsley P, Cowtan K. *Coot*: model-building tools for molecular graphics. *Acta Crystallogr D: Biol Crystallogr* **60**, 2126-2132 (2004).

25. Winn M, Isupov M, Murshudov GN. Use of TLS parameters to model anisotropic displacements in macromolecular refinement. *Acta Crystallogr D: Biol Crystallogr* **57**, 122-133 (2001).

26. Laskowski RA, MacArthur MW, Moss DS, Thornton JM. *PROCHECK*: a program to check the stereochemical quality of protein structures. *J Appl Crystallogr* **26**, 283-291 (1993).

27. Davis IW*, et al. MolProbity*: all-atom contacts and structure validation for proteins and nucleic acids. *Nucleic Acids Res* **35**, W375-W383 (2007).

28. Berman HM*, et al.* The protein data bank. *Nucleic Acids Res* **28**, 235-242 (2000).

29. Thiele NA, Wilson JJ. Actinium-225 for targeted α therapy: coordination chemistry and current chelation approaches. *Cancer Biother Radiopharm* **33**, 336-348 (2018).

30. National Nuclear Data Center. https://www.nndc.bnl.gov.

31. Deblonde GJP*, et al.* Chelation and stabilization of berkelium in oxidation state +IV. *Nat Chem* **9**, 843-849 (2017).

32. Kelley MP*, et al.* Bond covalency and oxidation state of actinide ions complexed with therapeutic chelating agent 3,4,3-LI(1,2-HOPO). *Inorg Chem* **57**, 5352-5363 (2018).

33. Zielińska B, Bilewicz A. The hydrolysis of actinium. *J Radioanal Nucl Chem* **261**, 195-198 (2004).
